# Sediment-Water Interfaces as Traps and Sources of Microplastic Fragments and Microfibers—Insights from Stream Flume Experiments

**DOI:** 10.1021/acsestwater.5c00643

**Published:** 2025-10-29

**Authors:** Uwe Schneidewind, Holly A. Nel, Jennifer Drummond, Anna Kukkola, Nicolai Brekenfeld, Andrew J. Chetwynd, Ben C. Howard, Valerie Ouellet, Katie Reilly, Mohammad Wazne, Chang Li, Iseult Lynch, Gregory Sambrook-Smith, Stefan Krause

**Affiliations:** † School of Geography, Earth & Environmental Sciences, 1724 University of Birmingham Edgbaston, Birmingham B15 2tt, U.K.; ‡ Université de Lyon, Université Claude Bernard Lyon 1, CNRS, ENTPE, UMR 5023 LEHNA, Villeurbanne F-69622, France; § Key Laboratory of Integrated Regulation and Resource Development of Shallow Lakes, Ministry of Education, College of Environment, Hohai University, Nanjing 210098, P. R. China; ∥ Birmingham Institute of Sustainability and Climate Action, University of Birmingham Edgbaston, B15 2tt Birmingham, U.K.

**Keywords:** microplastic particles, microfibers, deposition, resuspension, hyporheic sediments, turbulence, flume mesocosms

## Abstract

Microplastic pollution has been found to negatively impact water quality and ecosystem health in numerous riverine environments at different spatial and temporal scales. However, many of the underlying principles controlling microplastic transport and retention mechanisms are still poorly understood. Here, we study the deposition behavior of nylon fibers and fragments (small and large) in flow-controlled stream flume experiments with gravel or mixed sediment. We use a stochastic modeling approach and Latin hypercube sampling to optimize the parameters describing microplastic deposition and resuspension and relate deposition rates to settling rates calculated using Stoke’s law. Our experiments show that lower streamflow velocity leads to faster microplastic deposition, an effect that is shape-dependent and more pronounced for fibers. In experiments with similar flow velocity, large fragments were more quickly deposited in flumes containing gravel compared to mixed sediment. Stoke’s settling rates and model-based deposition rates can differ by several orders of magnitude, especially for fibers. For our flume experiments, these differences are attributed to transitional and turbulent flow near the streambed. Results emphasize that microplastic net deposition and near-bed transport cannot be well described by Stoke’s law. Results will further our understanding of microplastic fate and transport in riverine environments.

## Graphical abstract

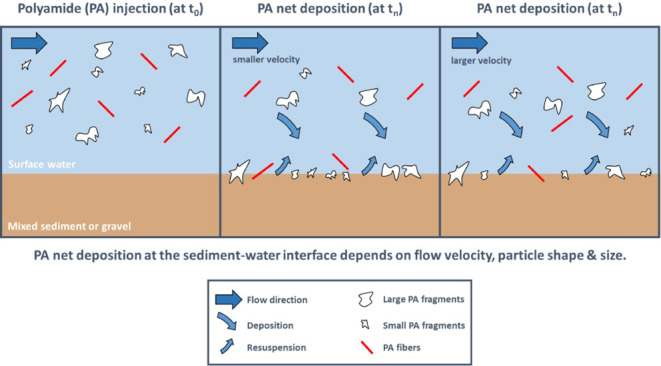

## 1 Introduction

Managing the major global challenge of environmental plastic pollution^1,2^ and its potential detrimental impacts on environmental and human health^3–5^ requires a sound understanding of the mechanisms controlling plastic distribution. This is particularly needed for microplastic particles (MP, 1–5000 μm), which have been detected in high concentrations in river systems globally.^6–8^ MP come in different shapes, with frequent shape descriptors used throughout literature being fibers, fragments and spheres.^9^ Fibers and fragments have been identified as the most common MP shapes in riverine networks, with fibers often related to input from domestic wastewater, tourism and fishing, while fragments mainly originate from nearby industrial areas, agricultural activity, wastewater effluent or the degradation of larger mismanaged plastic items.^10^

MP in rivers have been shown to adversely impact aquatic food chains, freshwater biota and ecosystem services.^11–14^ Although rivers represent fundamental pathways for MP to be transported from terrestrial and freshwater environments toward oceans^15,16^ they can also act as long-term sinks, delaying this process.^17^ During their passage through riverine environments, MP can undergo significant alteration and experience deposition, immobilization, and remobilization.^18^ Yet, while an increasing number of studies is focusing on uncovering the spatial and temporal occurrence of MP in rivers and river networks,^19–26^ it remains largely unknown how the shape and size of MP affect their transport and retention behavior.

The physicochemical properties of MPs and the associated hydrodynamic forcing play a decisive role in MP transport in riverine systems.^27–30^ Three general modes of MP transport in rivers can be distinguished, (i) floating at or near the surface of the water column, (ii) suspended transport and (iii) bedload transport. It has been argued that due to their distinct shape and density properties, the downstream transport of MP can differ substantially from that of inorganic sediment particles.^31^ Recent research on MP settling and rising velocities in the aquatic environment^32–34^ has shown that gravitational settling represents an important mechanism for MP transport through the water column, and that for MP with a density higher than water, gravitational settling should eventually lead to their deposition on the streambed. Other studies have found substantial concentrations of low-density MP also present in streambeds, which has been attributed to the occurrence of turbulent flow patterns close to the streambed surface and hyporheic exchange processes (i.e., mixing of surface and shallow subsurface water in the streambed) forcing MP into porous streambed sediment.^17,35–37^ However, how the relative impact of these different depositional mechanisms is affected by the strong contrasts in MP shapes and sizes found in river systems remains largely unknown.

It is challenging to systematically study the impacts of variable MP fate and transport processes for the wide range of existing MP properties and the diversity of naturally occurring hydrodynamic conditions. Therefore, controlled lab experiments such as in columns and stream flumes represent viable approaches for systematic testing. While column experiments can be helpful in uncovering vertical transport processes at small scale,^38–43^ they provide limited value for the study of MP deposition and resuspension (erosion) near the streambed–surface water interface since important drivers such as local hydrodynamic pressure differences, turbulence and surface water flow^37,44^ cannot be properly simulated. Thus, stream flume experiments have become a valuable tool for studying riverine MP transport under more controlled conditions. Previous stream flume experiments have studied the resuspension of various MP types from sediment beds with differences in their shape, size, and density,^45^ MP accumulation in different benthic organisms as well as sediment^46^ and lacustrine food webs,^47^ bedform-dependent microplastics transport,^48^ and potential MP retention of seagrass meadows.^49^

Here, we use stream flume experiments to discuss the depositional behavior of nylon (polyamide-6 or Nylon-6, hereafter referred to as nylon) fragments and fibers at the streambed–surface water interface. We hypothesize that near streambeds, gravitational forces alone cannot describe the deposition of the nylon particles sufficiently due to nonlaminar flow patterns and hyporheic exchange. Nylon is commonly used in the textile and automotive industries and has been identified as one of the major fibrous MP in wastewater effluent potentially released to rivers.^50^ It has also shown significant adsorption capacity and can act as a vector for pharmaceuticals and other contaminants in river water.^51^ Controlled flume settings are used to study the mechanisms controlling MP deposition and resuspension, depending on particle shape (i.e., fragments versus fibers) and size, flow velocity (4.9–9.2 cm s^–1^), and streambed sediment type (i.e., gravel versus heterogeneously mixed sediment). Flume experiments are combined with stochastic modeling to assess the effect of gravitational settling (as represented by the Stokes equation) compared to processes occurring near the streambed causing the exchange of MP between the water column and sediment, and thus to analyze the dominant transport mechanisms in river systems.

## 2 Materials and Methods

### 2.1 Microplastic Particles

This study analyzes the transport behavior of nylon fragments and fibers with a density of about 1.1 g·cm^–3^. Clear nylon pellets (Resinex Ltd., UK) were milled and sieved to obtain two distinct size fractions of fragments, from hereafter called small and large fragments (see Supporting Information for more details on fragment production). Laser diffraction was used on a subsample of both size fractions to determine the actual size distribution of the fragments (Figure S1) with high precision, employing a Mastersizer 2000 (Malvern Panalytical). 98.8% of MP in the group of small fragments were within 105–417 μm while 97.3% of the group of large fragments were between 275–832 μm in size (longest length). The particle lengths of the most common fractions (peaks of curves in Figure S1) were 182 μm (16.6%) and 479 μm (18.9%) for small and large fragment fractions, respectively. Scanning electron microscope (SEM, Phillips XL30 FEG ESEM) images taken of several fragments showed variation in particle size, surface roughness, roundness, and sphericity (Figure S2A,B), which was likely caused by their creation with the ball mill. Average particle masses were determined from Mastersizer data, with the small fragment particle mass being 8.00 × 10^–6^ g, and the large fragment mass being 8.22 × 10^–5^ g after applying a correction factor (see Section S1 for mass calculations). Red nylon fibers (1.7 dtex, defined as the mass in gram per 10,000 m or 0.17 g·km^–1^ fiber) commonly used in the automotive industry as filling material were obtained from Flock Depot GmbH (Germany). The fibers had been obtained precut to a length of 500 μm with a diameter of about 14 μm (according to company information, own SEM images confirmed 13–14.6 μm, Figure S2C,D). From the diameter and length of a single fiber, its average mass was determined as 8.50 × 10^–8^ g (see Section S1 for further details).

### 2.2 Flume Setup

Fifteen fiberglass recirculating flumes (Figure 1) were used to conduct stream flume experiments with the nylon MP. Before use, flumes were thoroughly cleaned with a power washer. Six different experimental set-ups were run in duplicate (Table 1), receiving either nylon fibers or fragments (small or large). Flumes were filled with either medium gravel (10–20 mm grains) or a commercial sediment mix (d_50_ = 0.66 mm) containing 71.2% sand, 21.7% fine gravel and 7.1% silt as determined by dry sieving (Section S2 and Figure S3). Sediment height in the gravel flumes was 4–5 cm, while sediment height in flumes containing the sediment mix was 2–3 cm. Three additional flumes were set up as control flumes without any initial MP input but containing gravel, mixed sediment or only water, respectively.

**Table 1:** Experimental Setup Showing Flume Identifier, Sediment Type, Microplastic (MP) Shape, Size and Mass Input as Well as Average Water Level on Top of the Sediment, Average Flow Velocity and the Average Time of Recirculation (Rec_Time) *t*_*r*_. G = Gravel Flume, M = Mixed Sediment Flume, FI = Fiber Flume, FL = Large Fragment Flume, FS = Small Fragment Flume, C = Control Flume

flume	Sediment	MP shape	MP size [μm]	MP input [g]	Average water level [cm]	Average velocity [cm s^–1^]	Average rec_time [s]
GFI1	gravel	fiber	500	3	6.8	8.0	49.8
GF12	gravel	fiber	500	3	6.7	5.9	67.4
MFI1	mixed	fiber	500	3	9.3	9.2	43.3
MF12	mixed	fiber	500	3	9.8	4.9	81.6
GFL1	gravel	fragment	large	10	6.8	7.6	52.4
GFL2	gravel	fragment	large	10	7.3	6.7	59.4
MFL1	mixed	fragment	large	10	9.2	6.8	59.1
MFL2	mixed	fragment	large	10	9.1	7.4	54.3
GFS1	gravel	fragment	small	5	7.2	7.4	53.8
GFS2	gravel	fragment	small	5	6.7	5.7	69.8
MFS1	mixed	fragment	small	5	9.5	7.0	56.9
MFS2	mixed	fragment	small	5	9.2	8.3	48.2
GC	gravel	NA	NA	0	6.1	7.7	51.7
MC	mixed	NA	NA	0	9.6	7.5	53.3
C	water only	NA	NA	0	8.3	12.2	32.7

**Figure 1:**
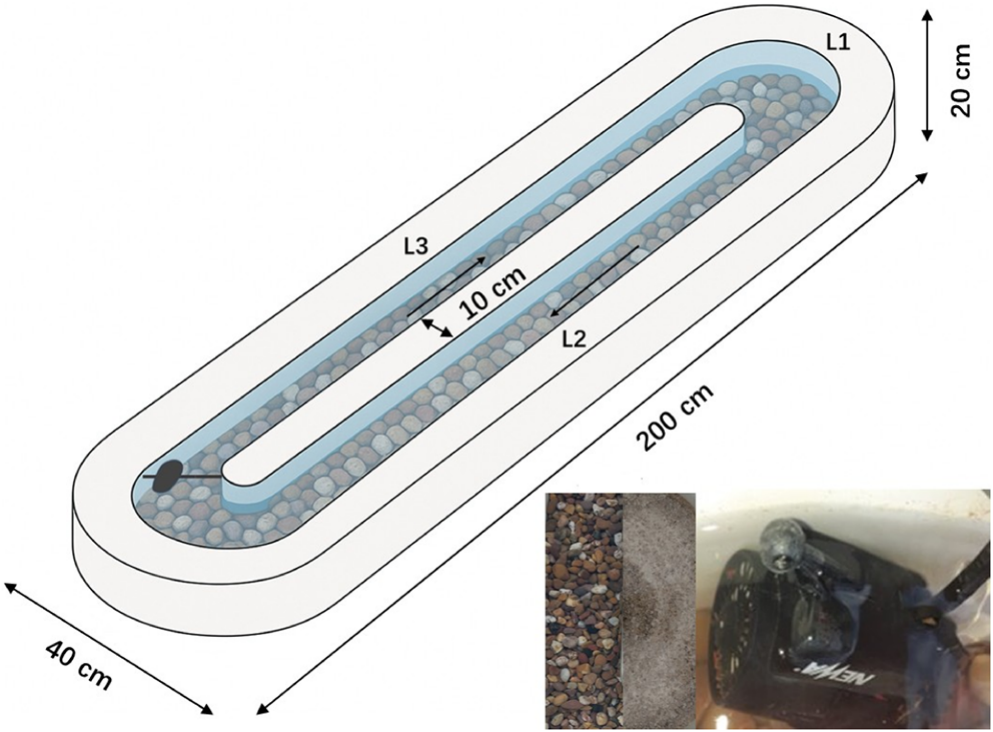
Schematic of a fiberglass recirculating flume used in this study. Locations L1, L2 and L3 indicate microplastics sampling points as well as locations of water level and flow velocity measurements. The arrows indicate the flow direction. Small inlay pictures show gravel, mixed sediment and aquarium pump used in the experiment.

Each flume was then filled with 45 L of groundwater obtained from an on-site borehole and connected to a single aquarium pump (Newa Wave, Italy). Afterward, flumes were left for several days covered with a blue Polytuf Medium Duty Tarpaulin (MAYO), composed of a UV resistant and waterproof, woven high density polyethylene (Section S2 and Figure S4) to avoid contamination by airborne particles. Prior to the start of the experiment, the water level (Table 1, Section 2 and Table S1) was measured at three locations in each flume (L1–L3, Figure 1) using a measuring stick. Flow velocity (Table 1, Section 2, and Table S2) was obtained at the same locations using an electromagnetic flowmeter (Valeport EM 801) by moving the flowmeter slowly up and down between 20% and 80% of the water column and averaging measurements over 30 s. The average water level on top of the sediment bed ranged from 6.7 to 7.3 cm in the gravel flumes and from 9.1 to 9.8 cm in mixed sediment flumes (Table 1). The observed minor water level variations within the flumes were attributed to nonuniform sediment packing or slight tilts at flume axes.

### 2.3 Flume Experiments

Nylon fragments were stained with Nile red dye before injection into the flumes, which resulted in their pink coloration. As such, both prestained fragments and red fibers were easily distinguishable from any other particles, including other potential MP that may have originated from sediment or water sources. Fragments and fibers were dispersed in a glass beaker containing 2.5 L of DI water using a glass rod for about 10 min. Afterward, they were rinsed into the respective flumes (now containing 47.5 L of water each). Fiber flumes then contained 3 g of red fibers per 47.5 L of water, while fragment flumes contained 5 g of small fragments or 10 g of large fragments per 47.5 L at time *t* = 0.

Each flume was sampled for MP at three locations (Figure 1, at the same position where flow velocity had been measured) at <1, 15, 30, 45, 60, 75, 90, 105, 120, 180, and 240 min after particle injection. At each location and per time point, one sample (aliquot) was collected in a 20 mL glass vial that was moved vertically upward through the entire water column. Each aliquot was filtered over a Whatman GF/D glass fiber filter (GE Healthcare and Life Sciences; 47 mm diameter, mesh size of 2.7 μm) immediately after sampling, and filters were stored in Petri dishes before analysis. Glassware and filtration systems were frequently washed and rinsed with DI water to avoid cross-contamination between samples. Control flumes were only sampled at the start and end of the experiment.

MP on GF/D filters were counted with a Zeiss Stemi 2000 stereo microscope using bright light (1.6× magnification for fragments and 1.6–5.0× for fibers). This was sufficient as the color of the target fibers (red) and fragments (pinkish) was known and target particles were easily identifiable. The few other potential plastic particles encountered on the filters were blue (tarpaulin flume cover material) and dark (from clothing of participants). The exact counting procedure is described in the SI (Section S3). MP counts were then corrected by averaged counts from the control flumes. Particle numbers were combined with mass information (see Section S1 for calculations) to determine the normalized concentration of MP retained in the water column at each sampling time.

### 2.4 Calculation of Microplastic Particle Settling

MP settling experiments are frequently performed in vertical columns using no-flow conditions. As such particle settling could be well described using Stoke’s law by including shape-dependent drag forces.^34,52^ However, MP in the streamwater column are not only subject to gravitational and drag forces but they are also impacted by the moving water column, which leads to particle advection and dispersion in the downstream direction.^31^ Together, these define MP deposition on and resuspension from the streambed (flume sediment layer). Here we compared observed and theoretical MP settling. Theoretical MP settling was calculated as described in Drummond et al.^37^ using Stokes Law, given as

1 $$G_{MP}=\frac{V_{s}}{d}$$

with *G*_MP_ [T^–1^] as the particle settling rate, *V*_s_ [LT^–1^] as the particle settling velocity (Stokes velocity) and *d* [L] as the flume-specific average water level above the sediment bed, which was derived from initial measurements in each flume (Table 1). *V*_s_ was obtained following Dietrich^53^ and Drummond et al.^37^ through

2 $$V_{s}={\left(\frac{\rho_{MP}-\rho_{w}}{\rho_{w}}g\vartheta w_{*}\right)}^{1/3}$$

with *ρ*_MP_ and *ρ*_w_ [ML^–3^] as the density of MP (here nylon) and water, respectively, *g* [LT^–2^] as the acceleration due to gravity, ϑ [L^2^T^–1^] as the kinematic viscosity of water (about 1.3 mm^2^ s^–1^ at 10°C which was the average ambient temperature during our experiments) and $w_{*}$ [-] as the dimensionless settling velocity that is based on the dimensionless particle diameter $D_{*}$ [-]. It is calculated following Dietrich^53^ as

3 $$w_{*}=1.71\times 10^{-4}D_{*}^{2};D_{*}<0.05$$

4 $$\log w_{*}=-3.76715+1.92944\left(\log D_{*}\right)-0.009815{\left(\log D_{*}\right)}^{2}-0.00575{\left(\log D_{*}\right)}^{3}+0.00056{\left(\log D_{*}\right)}^{4};,0.05<D_{*}<5\times 10^{9}$$

Equation 3 is applicable to near-spherical particles at small $D_{*}$ when significant viscous forces exist, while eq 4 is best used for spherical particles with increasing $D_{*}$ where the viscous resistance of the fluid becomes less important.^53^ The dimensionless particle diameter was deduced from the nominal particle diameter *D*_*n*_ [L] of a sphere of equivalent volume following Dietrich^53^ via

5 $$D_{*}=\left(\frac{\rho_{MP}-\rho_{w}}{\rho_{w}}\right)g\frac{D_{n}^{3}}{\vartheta^{2}}$$

Here we calculated two settling velocities and settling rates for comparison. One set is based on spheres of equivalent particle volume, while the second set uses *D*_*n*_ values based on laser diffraction (fragments) and SEM images (fibers). Section 2.1 and Table S3 (Section S4) provide more information.

Previous studies dedicated to particle settling in general and MP settling in particular have determined that the shape of a particle can have a significant impact on its settling velocity.^32,34,53–55^ Irregularly shaped particles tend to be most stable when the surface area moving in the direction of fall is at a maximum, which means that they must overcome greater friction of the water against the particle surface, which reduces their settling velocity. An irregular particle shape also increases the likelihood of particle instability in the water column due to e.g., rotation or oscillation which in turn decrease the vertical settling velocity. Here, this is especially relevant for the nylon fibers that have a true sphericity ψ of only 0.393 (see Section S1 for calculation). To account for irregularly shaped particles, a variety of shape factors are proposed by previous studies that can be used to correct estimates of settling velocity.^32,53,56,57^ For comparison, following Dietrich^53^ we used a revised method to estimate *V*_s_ for fibers that is based on the Corey shape factor CSF (0.1673, Section S4 for calculations) and the Janke geometric shape factor term *E* (calculations , Section S4) as follows

6 $$V_{s}=\frac{1}{18}\frac{gD_{n}^{2}}{\mu }\left(\rho_{MP}-\rho_{w}\right)E^{0.28}$$

with *μ* [ML^–1^T^–1^] as the dynamic viscosity of water (about 1.31 × 10^–3^ N·s/m^2^ at 10°C). Table S3 (Section S4) provides an overview of the calculated Stokes settling velocities for the fragments and fibers as well as the parameters used in their calculation. As Stokes Law is strictly only valid for laminar flow conditions, we determined both the flume-specific Reynolds numbers Re for open channel flow as well as the particle Reynolds numbers Re_P_ as shown in eqs S4 and S5 in Section S4.

### 2.5 Modeling Microplastic Deposition and Resuspension

MP net deposition was quantified by simulating particle deposition *N*_dep_(*t*) [particles·T^–1^] and resuspension *N*_res_(*t*) [particles·T^–1^] rates due to hyporheic exchange and near-bed turbulent flow in the recirculating flumes with a mobile-immobile model. This model partitions particles between the water column (i.e., mobile zone) and sediment (i.e., immobile zone) using Continuous Time Random Walk (CTRW) theory following Roche et al.^58^ Particle concentrations *C*(*t*) [particles·L^-3^] are assumed to be spatially uniform in the water column, i.e., the water column is assumed to be well-mixed as shown in eq 7.

7 $$\frac{dC\left(t\right)}{dt}V_{f}=-N_{dep}\left(t\right)+N_{res}\left(t\right)$$

Here, *V*_f_ [L^3^] is the volume of water in the respective flume. This assumption seems reasonable for small Rouse numbers.^58^ In our experiments, simplified dimensionless flume-specific Rouse numbers are close to 1 following the equations outlined in Cowger et al.^44^

A full description of the model is provided in Section S5. Briefly, the average measured velocities of each flume were used as model input. For standard flow (baseflow) conditions, deposition and resuspension can be linked to two key parameters: (i) the deposition rate onto the sediment Λ [T^–1^], which is linked to *N*_dep_(*t*) and (ii) β [-], which is linked to *N*_res_(*t*). The latter is a parameter describing the likelihood of particle resuspension during baseflow, where β varies between 0 to 1 with values closer to 1 demonstrating a higher likelihood of resuspension. $\Lambda =\frac{v_{dep}}{d}$ can be considered as a depth-normalized deposition velocity, with *v*_dep_ [L·T^–1^] representing typical field deposition velocities.^59^

Time *t* [T] was normalized by the flume-specific recirculation time *t*_*r*_ [T] to be able to directly compare the deposition and resuspension parameter values between the flumes at varying flow velocity. Following the fitting procedure outlined in Drummond et al.^60^ (see Section S5) we used a Latin hypercube approach to sample the parameter space (*n* = 27,000 variations). Parameter sets were constrained to minimize the mean square error (θ̂) estimated between the model output and the measured MP concentrations in the water column.

### 2.6 Data Correction with Information from Blanks

For this study, a total of 426 GF/D filters (18 blanks, 144 fiber filters, 264 fragment filters) were analyzed using light microscopy. MP number concentrations of all three sampling locations per flume were then averaged, transformed to mass concentrations per flume volume (time-varying) and normalized to the respective flume-specific input concentrations. The control flumes (flumes 13–15, Table S4 in Section S6) showed traces of contamination with fibers (on average 1.36 fibers per 20 mL) and small fragments (on average 0.21 fragments per 20 mL) but no larger size fragments were observed. Cross-contamination was easily identified as target MP had been stained with Nile red (fragments) or were of a distinct red color (fibers). To account for errors due to cross-contamination, these average values were then used to correct MP number counts found in the other flumes. The median difference between corrected and uncorrected counts for fiber flumes was only 0.13% and only some of the samples taken at 180 min or later started to show differences above 1.0%. A similar analysis for fragments was not possible as the initial number of fragments injected into each flume is not precisely known. We attribute cross-contamination of control flumes to insufficient cleaning of some vials and glassware during our field sampling as well as to strong winds that might have blown some of the lighter (smaller) particles from experimental flumes that were located adjacent to the control flumes.

## 3 Results and Discussion

Figures 2 and S5 show the normalized mass-based concentration–time series for each flume over the course of the experiment, indicating that small fragments tend to be deposited fastest while fibers are generally deposited more slowly. Deposition follows an exponential decay function in flumes GFI1&2, MFI1&2 and GFL1&2 (Section S7 and Figure S5A–C), a logarithmic decay in flumes MFL1&2 (Figure S5D), and a power law function decay in all flumes containing small fragments (MFS1&2 and GFS1&2, Figure S5 E-F). While in all flumes except flume MFI1 the concentration–time curves approach zero, a few MP were detected in the water column even at the end of the experiment indicating a possible constant low-degree resuspension of MP from the streambed sediment. As these observations regarding particle deposition could be related to flow velocity in the flumes, particle shape and sediment type used, these parameters are discussed further in the subsequent sections.

**Figure 2:**
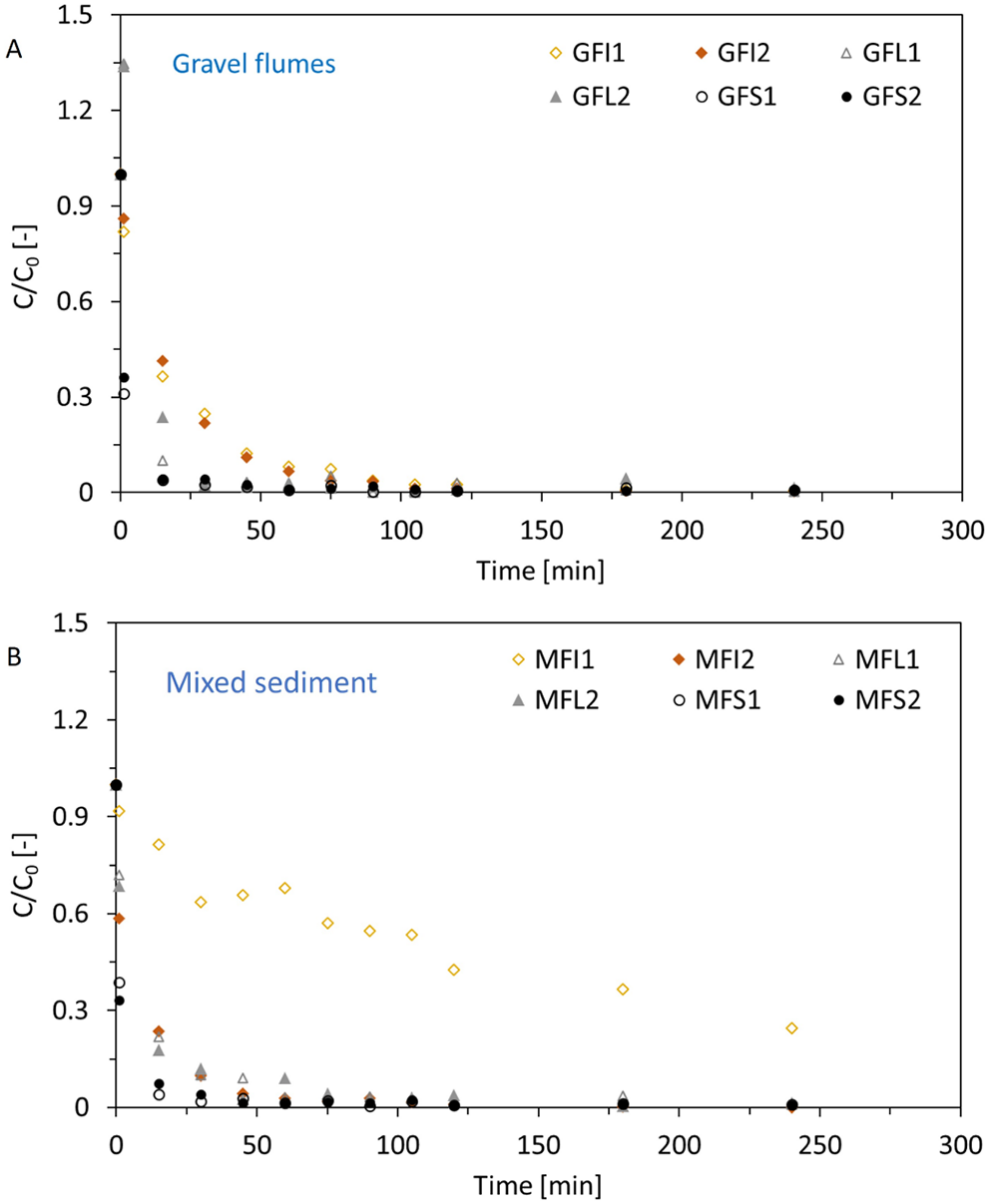
Normalized mass-based MP concentrations vs time for different flume setups (duplicates) categorized by sediment type (A—gravel flumes, B—mixed sediment flumes). Flume nomenclature: G = gravel flume, *M* = mixed sediment flume, FI = fiber flume, FL = large fragment flume, FS = small fragment flume. Flumes GFI, MFI and GFL follow exponential decay, flumes MFL follow a logarithmic decay and flumes GFS and MFS follow a power law decay. Trendlines and *R* ^2^ values are provided in Figure S5.

### 3.1 Impact of Flow Velocity, Particle Shape, and Sediment Type on Particle Settling

The average flow velocities in the flumes (Table 1) ranged from 4.9 cm s^–1^ (flume MFI2) to 9.2 cm s^–1^ (flume MFI1), with control flumes showing average velocities up to 12.2 cm s^–1^ (flume C). To better understand the impact that these flow velocity variations might have had on MP transport we divided the experimental time *t* by the flume-specific average time *t*_*r*_ it took a theoretical water parcel to recirculate (Table 1 and Figure 3) and compared for each shape (fiber, small fragment, large fragment) normalized concentrations to the number of recirculations for each sampling time. For this, it was assumed that the theoretical water parcel moved in a straight line downstream. The MP used in this experiment are expected to move slower than these theoretical water parcels as they experience particle drag and may collide with flume walls or other particles during their travel due to turbulence.

**Figure 3:**
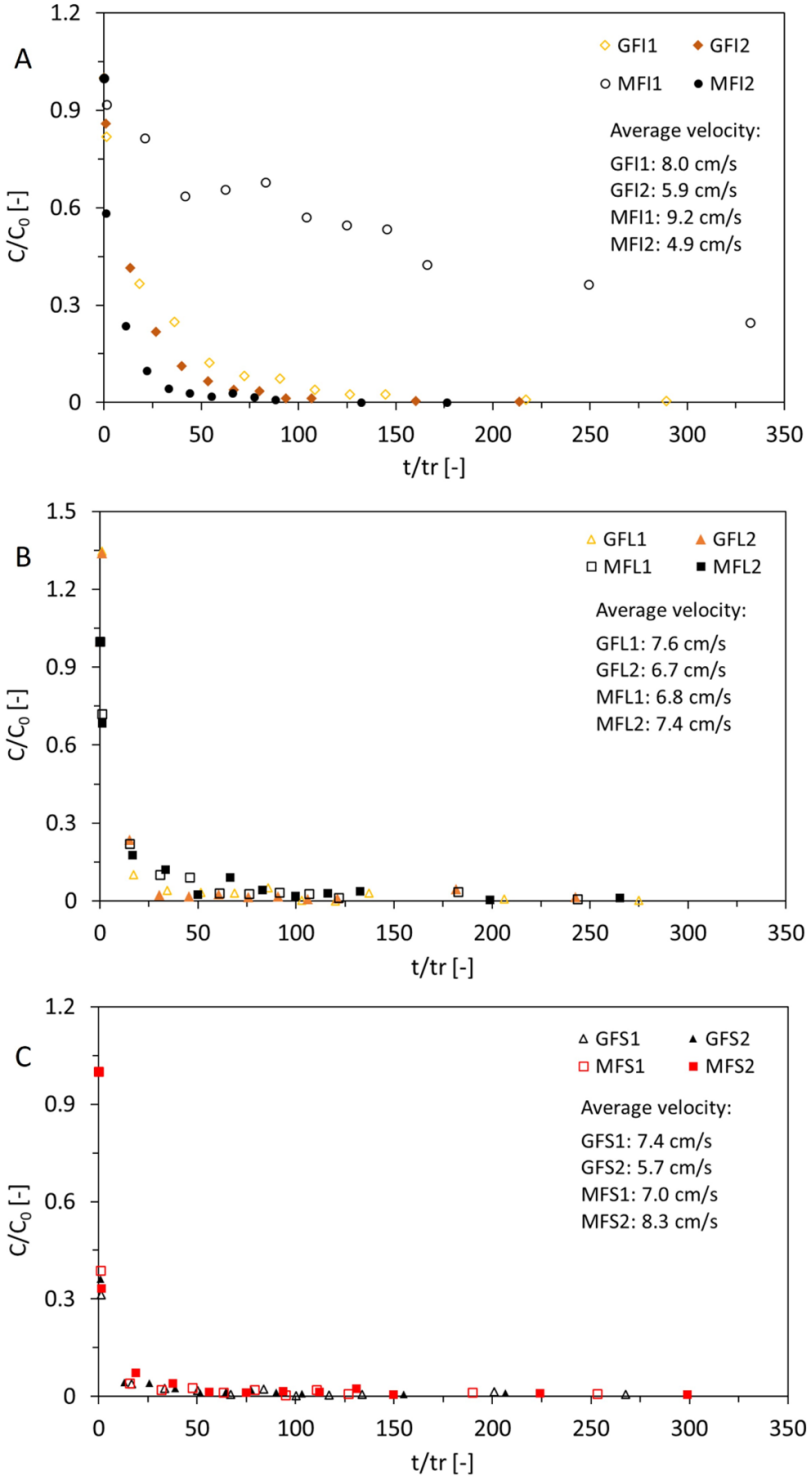
Normalized mass-based MP concentrations vs number of flume recirculations expressed as *t*/*t*_r_ where *t* is the experiment time and *t*_r_ is the average time water needs for one recirculation. A—fiber flumes, B—large fragment flumes and C—small fragment flumes for both sediment types. Flume nomenclature: G = gravel flume, M = mixed sediment flume, FI = fiber flume, FL = large fragment flume, FS = small fragment flume.

Figure 3 shows that in flumes with lower average flow velocity, particles deposit faster from the water column (i.e., less recirculations are needed) and a faster decrease of concentration can be observed. This effect is dependent on shape, being more pronounced for fibers (compare flumes GFI2 and MFI1&2 in Figure 3A) than for fragments (Figure 3B,C). This behavior is best seen during the early stage of the experiment (lower number of recirculations), compared to at a later stage when concentrations are generally lower. Figure 3B also demonstrates that in flumes with similar flow velocity (flumes GFL1 vs MFL2 and flumes GFL2 vs MFL1) the sediment type might exert additional influence on the deposition of MP of similar particle size and shape (here large fragments). In the early stages of the experiment (<50 recirculations), large fragments tended to deposit slightly faster in gravel flumes than in flumes containing mixed sediment. In flumes with similar velocity (e.g., flume GFI2 vs GFS2) but containing different MP shapes, fragments (flume GFS2) tended to deposit faster from the water column than fibers (flume GFI2).

In general, the impact of flow velocity on MP deposition rates in rivers and flumes is not yet well understood. While for stagnant water columns recent studies have described the effect of particle size, shape, density, water salinity and biofilm growth on vertical settling rates,^30,61,62^ river or open channel flow is often turbulent at least in some parts of the water column. This leads to additional transport processes acting on the MP, including additional drag, variations in the local flow velocity field, streambed roughness, hyporheic transport or local groundwater leading to MP erosion, and recent research has focused on developing models to describe some of these effects^63–65^

The shape of a particle can have a profound impact on its ability to move through the water column,^32,34^ with the particle aspect ratio (longest to shortest length) and the capability to reach its preferential orientation for downward transport being two main defining characteristics.^66,67^ The nylon fragments used in this study are more spherical compared to the fibers that resemble cylindrical objects and are less rigid. This leads to variations in the drag and lift forces exerted on fragments as compared to fibers, while secondary particle movements, such as lateral oscillation or rotation, reduce sinking velocities of fibers more strongly than of fragments.^34,68^ Consequently, numerous drag models have been developed for a variety of particle characteristics^69,70^ with some considering the differences between drag and lift when describing microfiber transport in aquatic systems.^69,71^

Recent studies have demonstrated that sediment suspended in the water column can lead to increased settling of both buoyant and nonbuoyant particles.^72^ MP and fine suspended sediment (higher density than MP) can form flocs or heteroaggregates^73^ with many small MP being found to migrate in flocs through freshwater systems.^74^ In our experiments occasional homoaggregation of nylon fibers was observed near the water surface. We also inspected the sediment bed after the experiment had concluded and the flumes had dried out, and found that in gravel flumes, the bed morphology was rather similar before and after the experiments. However, our observations here are purely qualitative as the experiment was designed to study MP deposition from the water column not focusing on disentangling the various processes relevant for determining particle infiltration depths in different sediment types. MP deposition on gravel beds commonly occurred in microtopographic depressions on the sediment surface or at borders between gravel grains (, Section S7 and Figure S6). In the mixed sediment flumes by contrast, often marked changes in bed microtopography occurred that led to MP deposition in patches along the longer sides of the flumes. Immediately after the pump, a zone was often encountered where sediment grains had been partially removed (Figure S7) due to the effect of the pump. These would then deposit somewhere along the flow path forming microbedforms (peaks and troughs) that could influence MP deposition patterns and drive advective pumping into the streambed.^75^

### 3.2 Particle Settling Rates and Reynolds Numbers

Theoretical particle settling rates *G*_MP_ (based on eq 1,2 and 6) are shown in Table S5 (Section S8). They were used to calculate a flume-specific expected time for particle deposition to be completed. For spherical particles with equivalent volumes (Table S5, data set A), large fragments need the least time for complete deposition (0.09 to 0.12 min), while small fragments require between 0.37 and 0.52 min and fibers require 8.83 to 12.89 min. When further including particle information obtained from mastersizer measurements (fragments) and SEM images (fibers) and shape factors for fibers in the calculations (eq 6 and Table S5 data set B), the expected times for complete particle settling increased to 0.10–0.13 min for large fragments, 0.61–0.86 min for small fragments and 309–451 min for fibers. These results are based on the assumption of a static water surface,^53^ and calculated settling velocities *V*_*s*_ (Section 2.4) ranging from 0.19 cm s^–1^ to 1.3 cm s^–1^ for fragments and 1.3 × 10^–2^ cm s^–1^ to 3.6 × 10^–4^ cm s^–1^ for fibers (Table S3, Section S4). Stoke’s settling velocities calculated in this study are in agreement with previous studies for static water surfaces.^61,76^ However, they contradict the particle settling behavior we observed (Figures 2, S3 and S5), which indicates nonlinear settling for all particle shapes influenced in part by the flume-specific flow velocities. In fact, in this experiment, fragments deposited much more slowly than predicted, with large fragments depositing even more slowly than small fragments. Vastly underpredicting the time to complete deposition could in part be a result of not including an additional shape factor for fragments, as was done for fibers as discussed by Goral et al.^77^ However, water flow in the flume (simulating streamflow) also has a profound impact on particle settling as can be seen when looking at the Reynolds numbers (Table S6). Although particle Reynolds numbers based on *V*_S_ assuming a static water surface were low (4.15 for large fragments, 0.26 for small fragments and 3.88 × 10^–4^ for fibers), the flume-specific Reynolds numbers for open channel flow ranged between 1553 and 2931, indicating rather transitional to turbulent flow. For higher Reynolds numbers the boundary layer thickness (i.e., the zone of contact between water and the MP) would quickly decrease and the water flow in the flumes adds an additional inertial component to the MP, increasing the impact of eddies and boils and keeping the MP in suspension for extended times. Such eddies resemble turbulences that are variable in space and time, and MP transport in turbulent flow conditions has been described by modeling advection and/or dispersion/diffusion components.^17,44^ At the sediment interface, MP have also potentially moved along the traction carpet via sliding, rolling or other bedload-specific transport processes.^31^ Additionally, over the course of the experiment, it was observed that MP can infiltrate into the streambed, especially into gravel. Although no measurements are available here, previous studies have discussed that infiltration of MP into streambeds occurs when the microplastic particle to grain size ratio is effectively *D* < 0.32.^38,78^ In our experiments, this is the case for the gravel flumes for all MP, but in the mixed sediment flumes, only small fragments seem to have the capability to infiltrate into the sediment bed (*D* = 0.28) while this capability is strongly limited for large fragments (*D* = 0.72). For fibers, infiltration into the mixed sediment depends on their orientation, with 0.05 ≤ *D* < 0.76.

### 3.3 Modeling Microplastic Particle Deposition and Resuspension as a Continuous Process

To better understand the discrepancy between theoretical times for complete particle deposition based on Stoke’s law and our observed experimental results, the stochastic mobile-immobile model described in Sections 2.5 and S5 was used to account for continuous particle deposition and resuspension during the flume experiments driven by near-bed nonlaminar flow. Modeling included curve fitting on the observed data, estimating deposition rate Λ [s^–1^] and resuspension parameter β [-], and determining the error associated with each parameter estimate. Figure 4A shows the modeling results for flume GFI1, results for the other flumes are shown in Figures S8–S11 (Section S8). Repeated curve fitting (*n* = 27,000) leads to a specific parameter combination for Λ and β for each model run. The parameter combinations with the least error (θ̂) are shown in Figure 4B (black and red dots), which demonstrates good parameter identifiability, and the best parameter combination was used to show the curve matching in Figure 4A.

**Figure 4:**
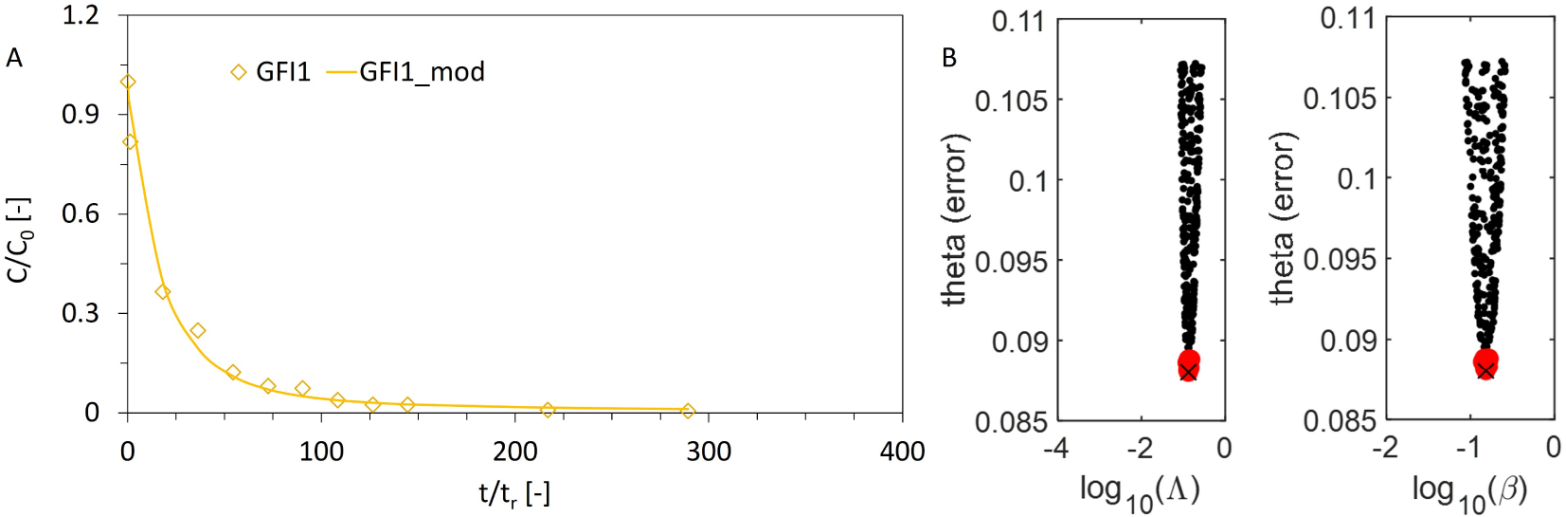
Modeling results for flume GFI1 (fibers) using a stochastic mobile-immobile model as outlined in the Supporting Information (Section S5). A—The model (continuous line) is optimized on the observed data (yellow diamonds). B—Model error vs parameter values. Black dots represent parameter values that meet a behavioral error threshold (top 1%) and red dots represent optimal parameter values (top 5% of the 1% shown or 0.05% of the total 27,000 runs). × symbolizes the best fit for the model curve in A, with the deposition rate Λ [s^–1^] resulting as 0.132 (standard deviation of 0.007), and the resuspension parameter β [-] as 0.153 (standard deviation of 0.008). Results for all other flumes are shown in Figures S8–S11 (semilog plots), and in Table S7.

Deposition rates Λ are highest for flumes with small fragments and cover a very narrow range as shown in Figure 5A and Table S7 (Section S8). Deposition rates for large fragments and fibers are generally lower than for small MP and cover a much wider range, which agrees well with our experimental observations, showing that those MP remain in the water column for longer. The resuspension parameter β, indicating the likelihood for MP resuspension from the sediment bed, is also highest for small fragments as compared to large fragments and fibers, but in general, likelihoods for resuspension at the given flume velocities are small. Values for β cover a much wider range for fiber and large fragment flumes (Figure 5), and especially for fibers, resuspension seems to play a more prominent role in particle settling as deposition rates are also low, which is especially visible for flume MFI1. Figure 5 shows that MP deposition and resuspension in our experiments are clearly shape-dependent, while the type of sediment simulating the streambed did not have such a distinct impact on MP settling as previously anticipated (Figure S12 and Section S8).

**Figure 5:**
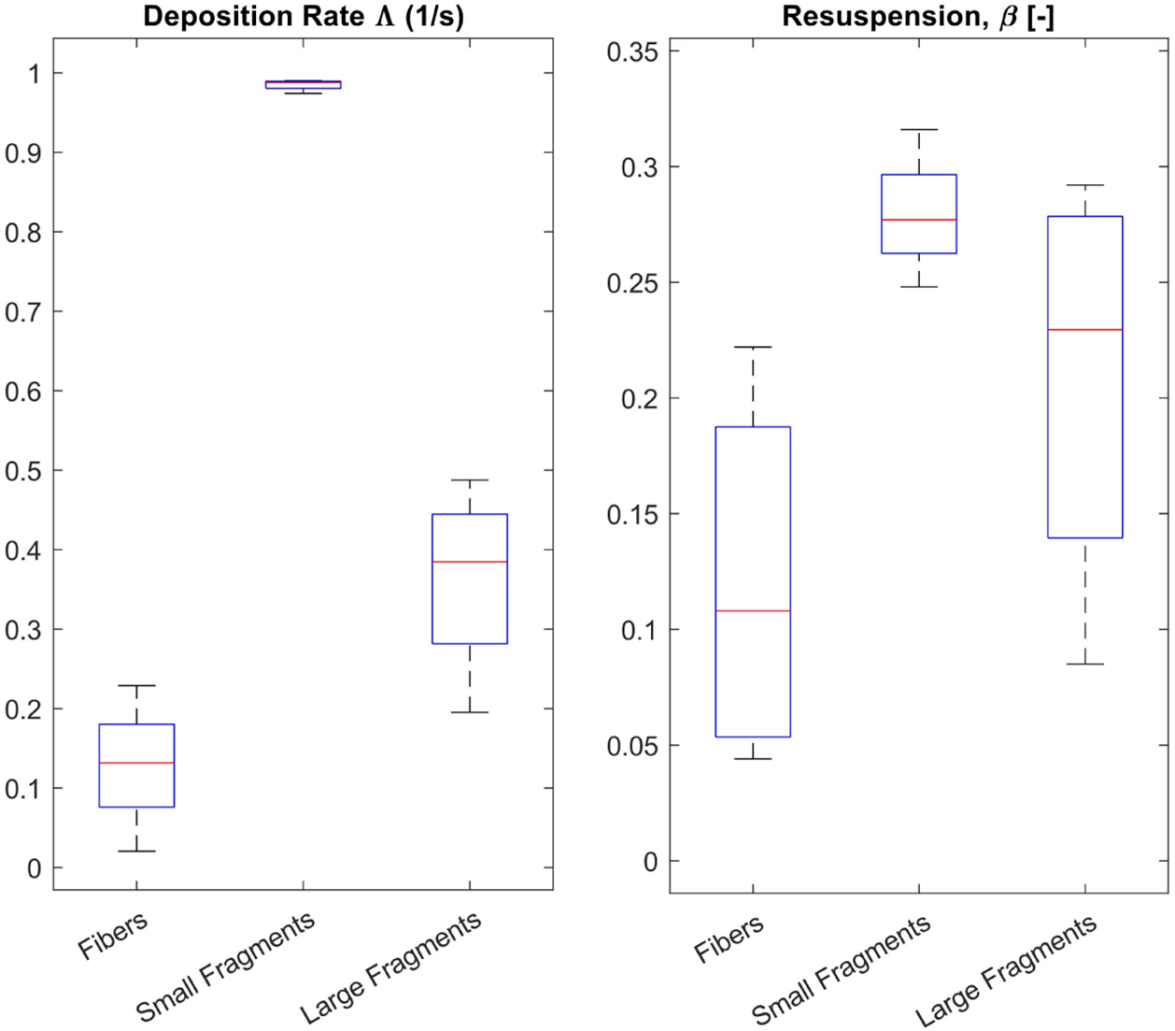
Box plots of deposition and resuspension rates grouped by MP shape (fiber, small fragment, large fragment). Statistics per group are based on best fit values (*n* = 4, Table S7, × in Figure S8) for each flume. The red line symbolizes the median, blue box 25th and 75th percentile, other lines show maxima and minima.

While theoretical particle settling rates *G*_MP_ are smaller for fragments and much smaller for fibers (up to almost 4 orders of magnitude, Table S5 and Table S7) than deposition rate Λ, quantifying net MP deposition by considering both particle deposition and resuspension provides a much more realistic explanation to our experimental observations.

Results of our flume experiments also suggest that nylon MP deposit out of the water column onto or partially infiltrate into the sediments. MP infiltration into the mixed sediment (d_50_ is 0.66 mm) was more likely for smaller MP (length of 182 μm) while larger MP and fibers had often just deposited on top of the sediment bed. In the gravel flumes, MP infiltration into the gravel bed was observed for all particle sizes. These observations agree with results from recent experiments that show that coarse-grained sediment with larger pore throat openings facilitate deeper advective transport of MP into the sediment, especially smaller sized ones^43,78,79^ before clogging or interactions with the sediment inhibit further MP transport.

### 3.4 Implications and Limitations

Our stream flume experiments show that the deposition of nylon MP depends on a variety of parameters such as particle size and shape as well as hydrodynamic conditions and streambed morphology. Nylon fragments in our experiments take a much longer time for complete deposition than would be expected using Stoke’s Law. On the other hand, the nylon fibers stay suspended in the water column for a longer period on average than fragments, most likely due to their cylindrical and elongated shape and their orientation. Conditions such as turbulent flow near the streambed or hyporheic exchange processes,^17,80^ can lead to an interplay of MP deposition and resuspension. Our results clearly highlight that MP transport near streambeds with microtopographic structures and transitional or turbulent flow should not be described by Stoke’s law.

While deposition and erosion patterns vary for MP of different density, shape, size, and surface structure (created e.g., by mechanical abrasion or biofouling), turbulent flow and hyporheic exchange processes are major drivers for downstream MP transport affecting their spatial footprint and fate, i.e., the physical and chemical alterations they experience under the specific conditions of their respective transport environments (benthic sediment, hyporheic sediment, near bed, water column).^31^ As such, turbulent flow and hyporheic exchange processes should be accounted for, e.g., when assessing local exposure and risks to downstream ecosystems due to MP pollution.^4,81,82^

Stream flume experiments such as those conducted here can be used to study target MP with known physicochemical characteristics and their interactions with other components at the sediment-water interface and in shallow streambeds. They can also serve as validation experiments in MP transport modeling studies.^83^ However, it should be noted that our findings come with certain limitations such as a relatively small flume depth (not more than 15 cm), assuming a well-mixed water column for modeling, or assuming a quasi-uniform flow field in the flume that allowed us to use an average velocity and neglect flume wall effects. Also, the flume design we used here seems not suitable to study additional processes that have been found to affect MP deposition and transport into deeper streambed sediment such as bedform celerity,^79^ channel reworking processes,^84^ additional groundwater upwelling or bioturbation.^81^

## 4 Conclusions

Microplastic particle deposition onto a streambed depends on particle characteristics (shape, size) as well as hydrodynamic conditions. Our experiments show that the net deposition rate of MP near the streambed requires the consideration of aspects such as particle resuspension (e.g., due to hyporheic exchange) and nonlaminar flow. Results will work toward improving our understanding of MP transport and fate in river systems and river networks.

## Supplementary Material



## Notes

Section 1: description of microplastic particles used in the experiment; Section 2: description of flume setup and flume experiments; Section 3: description of microplastic counting and microscopy procedure; Section 4: calculations of sphericity, shape factors, and Reynolds numbers; S5: detailed description of the model used; S6: results on particle counts; S7: results on particle concentrations; S8: results on particle deposition modeling (PDF)

## ∇

Center for Environment, Fisheries and Aquaculture Science, Pakefield Rd, Lowestoft, NR33 0HT, UK

## ○

Center for Proteome Research, Department of Biochemistry, Cell and Systems Biology, Institute of Systems, Molecular and Integrative Biology, University of Liverpool, Liverpool L69 7ZB, UK.

## ◆

Department of Civil and Environmental Engineering, Imperial College London, London SW7 2AZ, UK.

## ¶

Atlantic Salmon Federation, Chamcook, New Brunswick, E5B 3B1, Canada.

## #

Deceased in August 2025.

## References

[1] OECD. Global Plastics Outlook; OECD, 2022.

[2] Borrelle S.
B., Ringma J., Law K. L., Monnahan C. C., Lebreton L., McGivern A., Murphy E., Jambeck J., Leonard G. H., Hilleary M. A. (2020). Predicted growth in plastic waste exceeds efforts
to mitigate plastic pollution. Science.

[3] Krause S., Ouellet V., Allen D., Allen S., Moss K., Nel H. A., Manaseki-Holland S., Lynch I. (2024). The potential of micro-
and nanoplastics to exacerbate the health impacts and global burden
of non-communicable diseases. Cell Rep. Med..

[4] Krause S., Baranov V., Nel H. A., Drummond J. D., Kukkola A., Hoellein T., Smith G. H. S., Lewandowski J., Bonet B., Packman A. I. (2021). Gathering
at the top?
Environmental controls of microplastic uptake and biomagnification
in freshwater food webs. Environ. Pollut..

[5] Donisi I., Colloca A., Anastasio C., Balestrieri M. L., D’Onofrio N. (2024). Micro­(nano)­plastics: an Emerging
Burden for Human Health. Int. J. Biol. Sci..

[6] Li J., Liu H., Paul Chen J. (2018). Microplastics
in freshwater systems: A review on occurrence,
environmental effects, and methods for microplastics detection. Water Res..

[7] Singh J., Yadav B. K., Schneidewind U., Krause S. (2024). Microplastics pollution
in inland aquatic ecosystems of India with a global perspective on
sources, composition, and spatial distribution. J. Hydrol..

[8] Alimi O. S., Fadare O. O., Okoffo E. D. (2021). Microplastics
in African ecosystems:
Current knowledge, abundance, associated contaminants, techniques,
and research needs. Sci. Total Environ..

[9] Hartmann N. B., Huffer T., Thompson R. C., Hassellov M., Verschoor A., Daugaard A. E., Rist S., Karlsson T., Brennholt N., Cole M. (2019). Are We
Speaking the
Same Language? Recommendations for a Definition and Categorization
Framework for Plastic Debris. Environ. Sci.
Technol..

[10] Eerkes-Medrano D., Thompson R. C., Aldridge D. C. (2015). Microplastics in freshwater systems:
a review of the emerging threats, identification of knowledge gaps
and prioritisation of research needs. Water
Res..

[11] Capolupo M., Sorensen L., Jayasena K. D. R., Booth A. M., Fabbri E. (2020). Chemical composition
and ecotoxicity of plastic and car tire rubber leachates to aquatic
organisms. Water Res..

[12] Au S. Y., Bruce T. F., Bridges W. C., Klaine S. J. (2015). Responses of Hyalella
azteca to acute and chronic microplastic exposures. Environ. Toxicol. Chem..

[13] Kukkola A., Krause S., Lynch I., Smith G. H. S., Nel H. (2021). Nano and microplastic
interactions with freshwater biota – Current knowledge, challenges
and future solutions. Environ. Int..

[14] Ziajahromi S., Kumar A., Neale P. A., Leusch F. D. L. (2018). Environmentally
relevant concentrations of polyethylene microplastics negatively impact
the survival, growth and emergence of sediment-dwelling invertebrates. Environ. Pollut..

[15] Lebreton L. C. M., van der Zwet J., Damsteeg J.-W., Slat B., Andrady A., Reisser J. (2017). River plastic emissions to the world’s
oceans. Nat. Commun..

[16] Meijer L. J. J., van Emmerik T., van der Ent R., Schmidt C., Lebreton L. (2021). More than
1000 rivers account for 80% of global riverine plastic emissions into
the ocean. Sci. Adv..

[17] Drummond J. D., Schneidewind U., Li A., Hoellein T. J., Krause S., Packman A. I. (2022). Microplastic accumulation in riverbed
sediment via
hyporheic exchange from headwaters to mainstems. Sci. Adv..

[18] Hurley R., Woodward J., Rothwell J. J. (2018). Microplastic contamination of river
beds significantly reduced by catchment-wide flooding. Nat. Geosci..

[19] Mintenig S. M., Kooi M., Erich M. W., Primpke S., Redondo-
Hasselerharm P. E., Dekker S. C., Koelmans A. A., van Wezel A. P. (2020). A systems
approach to understand microplastic occurrence and variability in
Dutch riverine surface waters. Water Res..

[20] Mughini-Gras L., van der Plaats R. Q. J., van der Wielen P. W.
J. J., Bauerlein P. S., de Roda Husman A. M. (2021). Riverine microplastic and microbial community compositions:
A field study in the Netherlands. Water Res..

[21] Liu Y., Zhang J., Cai C., He Y., Chen L., Xiong X., Huang H., Tao S., Liu W. (2020). Occurrence
and characteristics of microplastics in the Haihe River: An investigation
of a seagoing river flowing through a megacity in northern China. Environ. Pollut..

[22] Frei S., Piehl S., Gilfedder B. S., Loder M. G. J., Krutzke J., Wilhelm L., Laforsch C. (2019). Occurence of microplastics in the
hyporheic zone of rivers. Sci. Rep..

[23] Sarkar D. J., Das Sarkar S., Das B. K., Manna R. K., Behera B. K., Samanta S. (2019). Spatial distribution of meso and
microplastics in the
sediments of river Ganga at eastern India. Sci.
Total Environ..

[24] Klein S., Worch E., Knepper T. P. (2015). Occurrence and Spatial Distribution
of Microplastics in River Shore Sediments of the Rhine-Main Area in
Germany. Environ. Sci. Technol..

[25] Kukkola A., Schneidewind U., Haverson L., Kelleher L., Drummond J. D., Smith G. S., Lynch I., Krause S. (2024). Snapshot Sampling May
Not Be Enough to Obtain Robust Estimates for Riverine Microplastic
Loads. ACS EST Water.

[26] Kelleher L., Schneidewind U., Krause S., Haverson L., Allen S., Allen D., Kukkola A., Murray-Hudson M., Maselli V., Franchi F. (2023). Microplastic
accumulation in endorheic
river basins – The example of the Okavango Panhandle (Botswana). Sci. Total Environ..

[27] Hoellein T. J., Shogren A. J., Tank J. L., Risteca P., Kelly J. J. (2019). Microplastic
deposition velocity in streams follows patterns for naturally occurring
allochthonous particles. Sci. Rep..

[28] Kooi M., Primpke S., Mintenig S. M., Lorenz C., Gerdts G., Koelmans A. A. (2021). Characterizing the multidimensionality
of microplastics
across environmental compartments. Water Res..

[29] Kukkola A., Runkel R. L., Schneidewind U., Murphy S. F., Kelleher L., Smith G. H. S., Nel H. A., Lynch I., Krause S. (2023). Prevailing
impacts of river management on microplastic transport in contrasting
US streams: Rethinking global microplastic flux estimations. Water Res..

[30] Mendrik F., Fernández R., Hackney C. R., Waller C., Parsons D. R. (2023). Non-buoyant
microplastic settling velocity varies with biofilm growth and ambient
water salinity. Commun. Earth Environ..

[31] Waldschläger K., Brückner M. Z. M., Carney Almroth B., Hackney C. R., Adyel T. M., Alimi O. S., Belontz S. L., Cowger W., Doyle D., Gray A. (2022). Learning
from natural sediments to tackle microplastics challenges: A multidisciplinary
perspective. Earth-Sci. Rev..

[32] Khatmullina L., Isachenko I. (2017). Settling velocity
of microplastic particles of regular
shapes. Mar. Pollut. Bull..

[33] Waldschläger K., Born M., Cowger W., Gray A., Schüttrumpf H. (2020). Settling and
rising velocities of environmentally weathered micro- and macroplastic
particles. Environ. Res..

[34] Waldschläger K., Schüttrumpf H. (2019). Effects of Particle Properties on
the Settling and
Rise Velocities of Microplastics in Freshwater under Laboratory Conditions. Environ. Sci. Technol..

[35] He B., Smith M., Egodawatta P., Ayoko G. A., Rintoul L., Goonetilleke A. (2021). Dispersal
and transport of microplastics in river sediments. Environ. Pollut..

[36] Nel H. A., Dalu T., Wasserman R. J. (2018). Sinks and sources: Assessing microplastic
abundance in river sediment and deposit feeders in an Austral temperate
urban river system. Sci. Total Environ..

[37] Drummond J. D., Nel H. A., Packman A. I., Krause S. (2020). Significance of Hyporheic
Exchange for Predicting Microplastic Fate in Rivers. Environ. Sci. Technol. Lett..

[38] Waldschlager K., Schuttrumpf H. (2020). Infiltration
Behavior of Microplastic Particles with
Different Densities, Sizes, and Shapes-From Glass Spheres to Natural
Sediments. Environ. Sci. Technol..

[39] Gao J., Pan S., Li P., Wang L., Hou R., Wu W.-M., Luo J., Hou D. (2021). Vertical migration of microplastics in porous media:
Multiple controlling factors under wet-dry cycling. J. Hazard. Mater..

[40] O’Connor D., Pan S., Shen Z., Song Y., Jin Y., Wu W.-M., Hou D. (2019). Microplastics undergo accelerated vertical migration in sand soil
due to small size and wet-dry cycles. Environ.
Pollut..

[41] Li M., Zhang X., Yi K., He L., Han P., Tong M. (2021). Transport and deposition
of microplastic particles in saturated porous
media: Co-effects of clay particles and natural organic matter. Environ. Pollut..

[42] Jiang Y., Yin X., Xi X., Guan D., Sun H., Wang N. (2021). Effect of
surfactants on the transport of polyethylene and polypropylene microplastics
in porous media. Water Res..

[43] Munz M., Loui C., Postler D., Pittroff M., Oswald S. E. (2024). Transport
and retention of micro-polystyrene in coarse riverbed sediments: effects
of flow velocity, particle and sediment sizes. Microplast. Nanoplast..

[44] Cowger W., Gray A. B., Guilinger J. J., Fong B., Waldschläger K. (2021). Concentration
Depth Profiles of Microplastic Particles in River Flow and Implications
for Surface Sampling. Environ. Sci. Technol..

[45] Waldschlager K., Schuttrumpf H. (2019). Erosion Behavior of Different Microplastic Particles
in Comparison to Natural Sediments. Environ.
Sci. Technol..

[46] de
Smit J. C., Anton A., Martin C., Rossbach S., Bouma T. J., Duarte C. M. (2021). Habitat-forming species trap microplastics
into coastal sediment sinks. Sci. Total Environ..

[47] Michler-Kozma D. N., Kruckenfellner L., Heitkamp A., Ebke K. P., Gabel F. (2022). Uptake and
Transfer of Polyamide Microplastics in a Freshwater Mesocosm Study. Water.

[48] Boos J.-P., Gilfedder B. S., Frei S. (2021). Tracking Microplastics Across the
Streambed Interface: Using Laser-Induced-Fluorescence to Quantitatively
Analyze Microplastic Transport in an Experimental Flume. Water Resour. Res..

[49] de
Los Santos C. B., Krång A.-S., Infantes E. (2021). Microplastic retention
by marine vegetated canopies: Simulations with seagrass meadows in
a hydraulic flume. Environ. Pollut..

[50] Sun J., Dai X., Wang Q., van Loosdrecht M. C.
M., Ni B.-J. (2019). Microplastics
in wastewater treatment plants: Detection, occurrence and removal. Water Res..

[51] Wagstaff A., Lawton L. A., Petrie B. (2022). Polyamide microplastics
in wastewater
as vectors of cationic pharmaceutical drugs. Chemosphere.

[52] Li C., Bai X., Krause S., Luo D. (2024). Prediction of vertical transport
of microplastics: Shape- and aging-dependent drag models. J. Hazard. Mater..

[53] Dietrich W. E. (1982). Settling
velocity of natural particles. Water Resour.
Res..

[54] Komar P. D., Reimers C. (1978). Grain Shape Effects on Settling Rates. J. Geol..

[55] Kowalski N., Reichardt A. M., Waniek J. J. (2016). Sinking rates of microplastics and
potential implications of their alteration by physical, biological,
and chemical factors. Mar. Pollut. Bull..

[56] Janke N. C. (1966). Effect
of shape upon the settling vellocity of regular convex geometric particles. J. Sediment. Res..

[57] Komar P. D. (1980). Settling
Velocities of Circular Cylinders at Low Reynolds Numbers. J. Geol..

[58] Roche K. R., Drummond J. D., Boano F., Packman A. I., Battin T. J., Hunter W. R. (2017). Benthic biofilm controls on fine particle dynamics
in streams. Water Resour. Res..

[59] Newbold J. D., Thomas S. A., Minshall G. W., Cushing C. E., Georgian T. (2005). Deposition,
benthic residence, and resuspension of fine organic particles in a
mountain stream. Limnol. Oceanogr..

[60] Drummond J., Schmadel N., Kelleher C., Packman A., Ward A. (2019). Improving
Predictions of Fine Particle Immobilization in Streams. Geophys. Res. Lett..

[61] Dittmar S., Ruhl A. S., Jekel M. (2023). Optimized
and Validated Settling
Velocity Measurement for Small Microplastic Particles (10–400
μm). ACS EST Water.

[62] Dittmar S., Ruhl A. S., Altmann K., Jekel M. (2024). Settling Velocities
of Small Microplastic Fragments and Fibers. Environ. Sci. Technol..

[63] Ijaz U., Baki A. B. M., Wu W., Zhang W. (2024). Settling velocity of
microplastics in turbulent open-channel flow. Sci. Total Environ..

[64] Akdogan Z., Guven B. (2025). Sensitivity analysis of a one-dimensional microplastic transport
model in turbulent rivers: Intrinsic properties and hydrodynamics. J. Environ. Manage..

[65] Akdogan Z., Guven B. (2024). Modeling the settling and resuspension of microplastics in rivers:
Effect of particle properties and flow conditions. Water Res..

[66] DiBenedetto M. H., Ouellette N. T., Koseff J. R. (2017). Transport of anisotropic particles
under waves. J. Fluid Mech.

[67] Van
Melkebeke M., Janssen C., De Meester S. (2020). Characteristics
and Sinking Behavior of Typical Microplastics Including the Potential
Effect of Biofouling: Implications for Remediation. Environ. Sci. Technol..

[68] Ji C., Zhang J., Liu G., Zhang Q., Shen X. (2024). A settling
velocity formula for irregular shaped microplastic fragments based
on new shape factor: Influence of secondary motions. Sci. Total Environ..

[69] Choi C. E., Zhang J., Liang Z. (2022). Towards realistic predictions of
microplastic fiber transport in aquatic environments: Secondary motions. Water Res..

[70] Francalanci S., Paris E., Solari L. (2021). On the prediction of settling velocity
for plastic particles of different shapes. Environ.
Pollut..

[71] Zhang J., Choi C. E. (2025). Towards A universal
settling model for microplastics
with diverse shapes: Machine learning breaking morphological barriers. Water Res..

[72] Mancini M., Serra T., Colomer J., Solari L. (2023). Suspended sediments
mediate microplastic sedimentation in unidirectional flows. Sci. Total Environ..

[73] Wang Z., Dou M., Ren P., Sun B., Jia R., Zhou Y. (2021). Settling velocity
of irregularly shaped microplastics under steady and dynamic flow
conditions. Environ. Sci. Pollut. Res..

[74] Wu N., Grieve S. W. D., Manning A. J., Spencer K. L. (2024). Flocs as vectors
for microplastics in the aquatic environment. Nat. Water.

[75] Boano F., Harvey J. W., Marion A., Packman A. I., Revelli R., Ridolfi L., Wörman A. (2014). Hyporheic
flow and transport processes:
Mechanisms, models, and biogeochemical implications. Rev. Geophys..

[76] Shen X., Lin M., Chong H., Zhang J., Li X., Robins P., Bi Q., Zhu Y., Zhang Y., Chen Q. (2024). Settling and rising
velocities of microplastics: Laboratory experiments and lattice Boltzmann
modeling. Environ. Pollut..

[77] Goral K. D., Guler H. G., Larsen B. E., Carstensen S., Christensen E. D., Kerpen N. B., Schlurmann T., Fuhrman D. R. (2023). Settling velocity of microplastic particles having
regular and irregular shapes. Environ. Res..

[78] Boos J.-P., Dichgans F., Fleckenstein J. H., Gilfedder B. S., Frei S. (2024). Assessing the Behavior of Microplastics
in Fluvial Systems: Infiltration
and Retention Dynamics in Streambed Sediments. Water Resour. Res..

[79] Peleg E., Teitelbaum Y., Arnon S. (2024). Exploring the influence of sediment
motion on microplastic deposition in streambeds. Water Res..

[80] Wazne M., Simon L., Krause S., Vallier M., Dendievel A.-M., Touchet C. M., Mourier B., Montagnac G., Mermillod-Blondin F. (2025). Microplastics storage at the sediment-water
interface
in a gravel-bed river: Importance of local hydro-sedimentary conditions
in downwelling, upwelling, and sedimentation zones. Water Res..

[81] Wazne M., Mermillod-Blondin F., Vallier M., Hervant F., Dumet A., Nel H. A., Kukkola A., Krause S., Simon L. (2023). Microplastics
in Freshwater Sediments Impact the Role of a Main Bioturbator in Ecosystem
Functioning. Environ. Sci. Technol..

[82] Sfriso A. A., Tomio Y., Juhmani A.-S., Sfriso A., Munari C., Mistri M. (2021). Macrophytes: A Temporary
Sink for Microplastics in
Transitional Water Systems. Water.

[83] Dichgans F., Boos J.-P., Ahmadi P., Frei S., Fleckenstein J. H. (2023). Integrated
numerical modeling to quantify transport and fate of microplastics
in the hyporheic zone. Water Res..

[84] Arnon S. (2025). Making waves:
Unraveling microplastic deposition in rivers through the lens of sedimentary
processes. Water Res..

