# Supplementary material for: Sediment-Water Interfaces as Traps and Sources of Microplastic Fragments and Microfibers—Insights from Stream Flume Experiments
Source: ACS ES T Water. 2025 Oct 29;5(11):6567–78. doi: 10.1021/acsestwater.5c00643 (PMC12624745; doi:10.1021/acsestwater.5c00643)
Supplement: Supplementary file 1 [file ew5c00643_si_001.pdf]

Supporting Information

Sediment-water interfaces as traps and sources of microplastic fragments and microfibers – Insights from stream flume experiments

Uwe Schneidewind[1,*], Holly A. Nel[1,a], Jennifer Drummond[1], Anna Kukkola[1,†], Nicolai Brekenfeld[1], Andrew J. Chetwynd[1,c], Ben C. Howard[1,d], Valerie Ouellet[1,e], Katie Reilly[1], Mohammad Wazne[1,2], Chang Li[3], Iseult Lynch[1,4], Gregory Sambrook-Smith[1], Stefan Krause[1,2,4]

[1]School of Geography, Earth & Environmental Sciences, University of Birmingham, Edgbaston, B15 2TT Birmingham, UK.

[2]Université de Lyon, Université Claude Bernard Lyon 1, CNRS, ENTPE, UMR 5023 LEHNA, Villeurbanne, France.

[3]Key Laboratory of Integrated Regulation and Resource Development of Shallow Lakes, Ministry of Education, College of Environment, Hohai University, Nanjing 210098, PR China.

[4]Birmingham Institute of Sustainability and Climate Action, University of Birmingham, Edgbaston, B15 2TT Birmingham, UK.

*Corresponding author: Uwe Schneidewind, u.schneidewind@bham.ac.uk, School of Geography, Earth & Environmental Sciences, University of Birmingham, Edgbaston, B15 2TT, Birmingham, UK.

[a]now at Centre for Environment, Fisheries and Aquaculture Science, Pakefield Rd, Lowestoft, NR33 0HT, UK.

[b]now at Centre for Proteome Research, Department of Biochemistry, Cell and Systems Biology, Institute of Systems, Molecular and Integrative Biology, University of Liverpool, Liverpool L69 7ZB, UK.

[c]now at Department of Civil and Environmental Engineering, Imperial College London, London SW7 2AZ, UK.

[d]now at Atlantic Salmon Federation, Chamcook, New Brunswick, E5B 3B1, Canada.

[†]Deceased in August 2025

Content:

- S1 – Description of microplastic particles used in the experiment
- S2 – Description of flume setup and flume experiments
- S3 – Description of microplastic counting and microscopy procedure
- S4 – Calculations of sphericity, shape factors, Reynolds numbers
- S5 – Detailed description of model
- S6 – Particle counts
- S7 – Particle concentrations
- S8 – Particle deposition

S1 – Microplastic particles (MP) used in the experiments

Clear nylon (PA-6, Nylon-6) pellets (Resinex Ltd., UK) were frozen at -80°C for at least 72 hours, after which the pellets were mechanically ground using a vibratory ball mill (Fritsch Micro Mill Pulverisette 0) and liquid nitrogen. After grinding, fragments were dry- and wet-sieved by hand using various stainless-steel sieves (150 µm – 600 µm range). Two distinct size fractions from hereafter called large and small fragments were oven-dried (50°C) and retained for the flume experiments. Laser diffraction was then used on a subsample of both fractions to determine the actual size distribution of the fragments by employing a Mastersizer 2000 (Malvern Panalytical) with MilliQ water as dispersant and the ultra-sonication probe for proper particle dispersion. We did not use any additional surfactant to further reduce surface tension and counteract potential aggregation.

Figure S1 shows the two particle size distribution curves obtained from laser diffraction. According to these distribution curves, 98.8 % of the small fragments fell within 105–417 µm while 97.3 % of the large particles were between 275–832 µm. The particle diameters of the most common fractions (peaks of curves in Figure S1) were 181.97 µm (16.60%) and 478.63 µm (18.94%), respectively. Particle diameters are nominal particle diameters D_S of spheres with equivalent volume of the actual measured fragments, thus potentially slightly overestimating any settling velocities for fragments that considerably deviate from spherical shape.

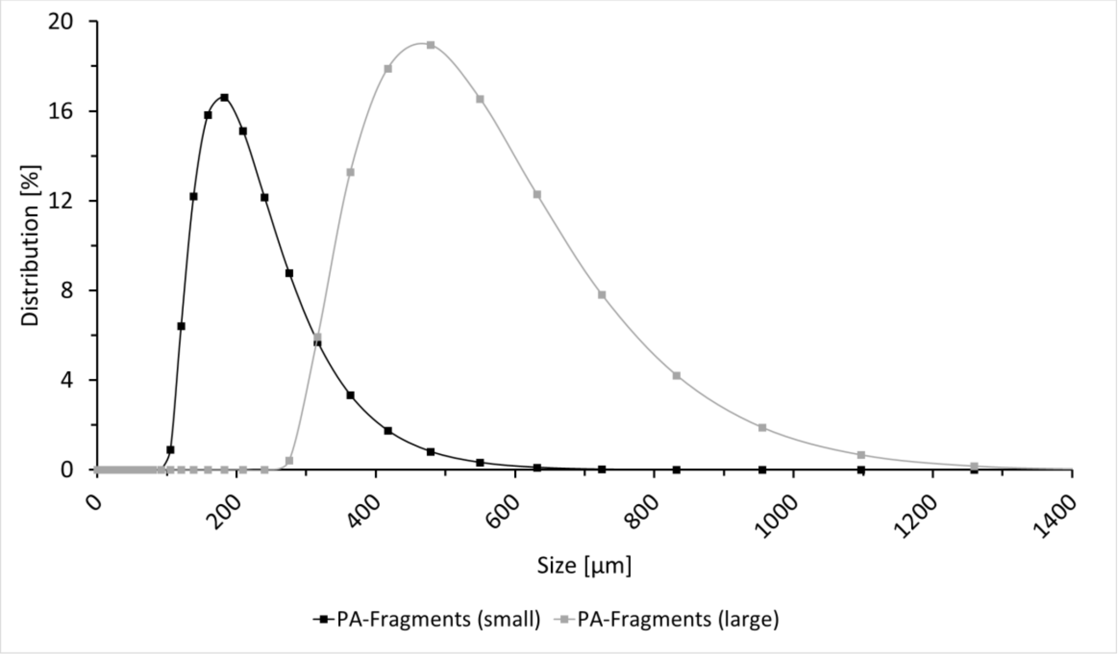

Figure S1: Size distribution of nylon fragments in two different size ranges obtained from laser diffraction. These two groups were then used in the flume experiments.

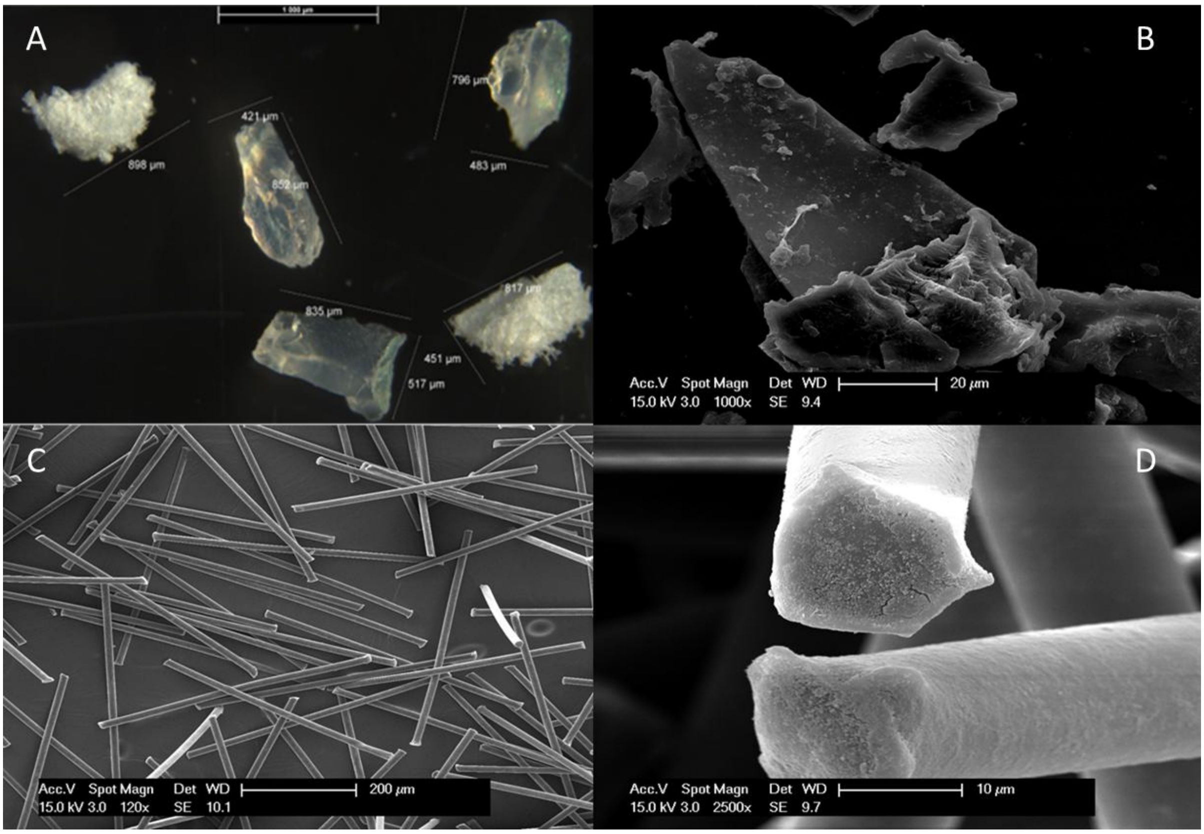

Figure S2: Nylon fragments (A-B) and fibers (C-D) used in the experiments. Pictures were taken with a scanning electron microscope (Phillips XL30 FEG ESEM). Particles were sputter-coated with platinum for imaging and images were acquired at 15 kV and 80-20,000× magnification. While fibers are all very similar, fragment surface structure, roundness and sphericity varied considerably due to the effect of the ball mill.

The mass of a fiber was determined by using the diameter information provided by the supplier, i.e., 14 µm and the fiber length of 500 µm. By assuming that these short and rigid fibers are basically cylinders, the mass of one fiber was determined as 8.50×10^{-8} g. Fragment masses are based on Mastersizer measurements of 100 particles. For the small fragments an average mass of 8.00×10^{-6} g was determined while that of an average large fragment was initially determined at 10.89×10^{-5} g. An additional experiment was performed to address the quality of the Mastersizer data. In this experiment, a pre-known mass of fragments stained with Nile Red following [1] was filtered onto Whatman GF/D filters and particles were then counted using a fluorescent microscope (Olympus MVX-ZB10) with a 1 x 0.25 N objective (MVPLAPO 1X, Olympus), U-M49002XL GFP filter cube (excitation filter: 470/40 nm, dichroic mirror: 495 nm high pass, emission filter: 525/50 nm) and with a 100 W mercury apo lamp light source. The experiment was conducted five times and by comparing fragment numbers actually counted on the GF/D filters with fragment numbers expected from the Mastersizer measurements we found that the average recovery rate was 132.6 % with a standard deviation of 9.3 %. As such we corrected the initial average mass of a large fragment obtained from the Mastersizer experiments accordingly, to the new value of 8.22×10^{-5} g, while for the small fragments a correction was not required.

S2 – Flume experiments

Flumes, similar to the ones used in our experiment had previously been utilized, e.g., to study the transformation of wastewater-derived organic contaminants [2], the impact of hyporheic exchange flow, bedform and microbial diversity on the fate of micropollutants [3, 4], and the impact of low flow and extended high temperatures on various riverine ecosystem functions [5]. Figure S3 shows the two types of sediment used in the experiments and the grain size curve of the mixed sediment obtained from dry sieving. For the sieving, 733.4 g of mixed sediment material (oven-dried at 105 °C for several days) was used. Sieve mesh sizes used were 8, 4, 2, 1, 0.5, 0.25, 0.125, and 0.063 mm. Material loss during sieving was 0.4 % mass.

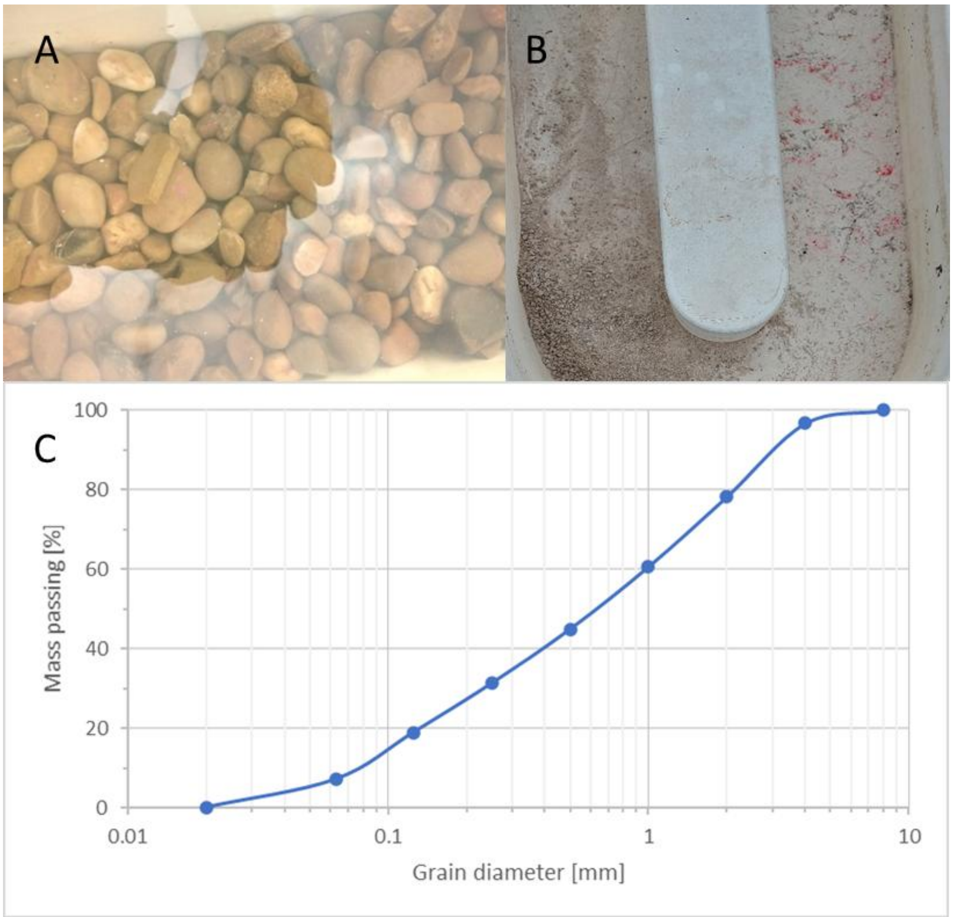

Figure S3: Sediment used in the flume experiments. (A) Gravel of 10-20 mm in size and (B) sediment mix containing sand, gravel and silt. (C) Grain size distribution curve of the sediment mix used in some of the flumes based on dry sieving. The sediment mix contained 71.2 % sand, 21.7 % fine gravel and 7.1 % silt according to ISO 14688-1:2017. The d_{50} is 0.66 mm.

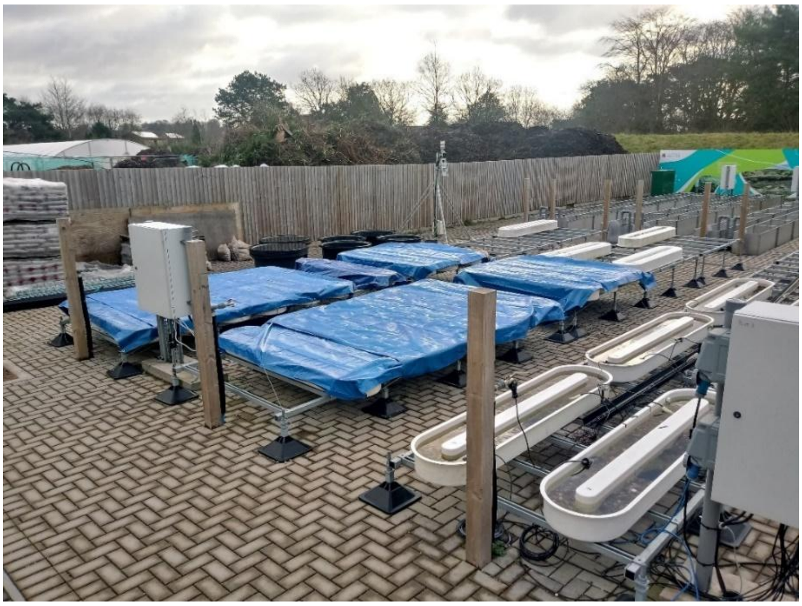

Figure S4: Recirculating flumes covered with Polytuf Medium Duty Tarpaulin (MAYO) before the experiment start and during times the flumes were not worked on. The cover provided protection from contamination with airborne particles.

Table S1: Water level height in the flumes on top of the sediment bed at three different sampling locations (L1-L3, as indicated in Figure 1), measured before the start of plastic injection. G = gravel flume, M = mixed sediment flume, FI = fiber flume, FL = large fragment flume, FS = small fragment flume, C = control flume.

Flume	Sediment	MP shape	L1	L2	L3	Average
			[cm]	[cm]	[cm]	[cm]
GFI1	gravel	fibre	6.5	7.0	7.0	6.8
GFI2	gravel	fibre	6.5	7.2	6.5	6.7
MFI1	mixed	fibre	9.0	9.5	9.5	9.3
MFI2	mixed	fibre	9.7	9.8	10.0	9.8
GFL1	gravel	fragment	7.5	6.5	6.5	6.8
GFL2	gravel	fragment	8.0	6.8	7.0	7.3
MFL1	mixed	fragment	9.0	9.5	9.0	9.2
MFL2	mixed	fragment	9.2	9.3	8.8	9.1
GFS1	gravel	fragment	7.5	6.5	7.5	7.2
GFS2	gravel	fragment	7.1	7.0	6.1	6.7
MFS1	mixed	fragment	9.5	9.5	9.5	9.5
MFS2	mixed	fragment	9.5	9.0	9.0	9.2
GC	gravel	blank	5.5	6.5	6.3	6.1
MC	mixed	blank	9.0	9.9	9.8	9.6
C	water only	blank	8.3	8.5	8.0	8.3

Table S2: Flow velocities and their standard deviations (STDEV) at three different sampling locations (L1-L3, as indicated in Figure 1) in the flumes obtained using a Valeport EM 801 electromagnetic flowmeter before particle injection. ND = no data. G = gravel flume, M = mixed sediment flume, FI = fiber flume, FL = large fragment flume, FS = small fragment flume, C = control flume.

	Sediment	MP shape	L1	STDEV - L1	L2	STDEV - L2	L3	STDEV - L3	Average
			[cm s^{-1}]	[cm s^{-1}]	[cm s^{-1}]	[cm s^{-1}]	[cm s^{-1}]	[cm s^{-1}]	[cm s^{-1}]
GFI1	gravel	fibre	9.0	0.6	8.6	0.5	6.5	0.9	8.0
GFI2	gravel	fibre	6.7	0.5	7.2	0.5	3.9	1.1	5.9
MFI1	mixed	fibre	9.1	0.8	10.4	0.7	8.2	0.8	9.2
MFI2	mixed	fibre	ND	ND	5.3	0.6	4.5	0.6	4.9
GFL1	gravel	fragment	9.7	0.9	8.4	0.7	4.8	0.7	7.6
GFL2	gravel	fragment	7.1	0.5	9.5	0.8	3.6	1.4	6.7
MFL1	mixed	fragment	4.7	1.1	7.7	0.7	7.9	0.8	6.8
MFL2	mixed	fragment	5.5	0.9	9.5	0.5	7.1	1.1	7.4
GFS1	gravel	fragment	8.2	0.8	6.3	0.6	7.8	0.8	7.4
GFS2	gravel	fragment	5.1	0.9	7.9	0.5	4.2	1.5	5.7
MFS1	mixed	fragment	4.2	1.2	8.8	0.4	8.1	0.9	7.0
MFS2	mixed	fragment	6.3	0.8	9.5	0.4	9.1	1.1	8.3
GC	gravel	blank	10.2	0.6	7.8	0.9	5.2	1.2	7.7
MC	mixed	blank	4.4	1.8	7.9	1.7	10.2	0.5	7.5
C	water only	blank	11.7	0.7	13.4	1.3	11.6	1.2	12.2

The highest average velocity was measured in flume 15 that contained only water. It is assumed that the sediment bed in the respective flumes reduced the flow velocity due to additional friction between water and sediment grains. Average velocities in the mixed sediment flumes were mostly slightly larger than in the gravel flumes, which is probably due to the more irregular packing of the latter and difference in grain roughness. We also found that for most flumes (except for flume 3) containing mixed sediment, the velocity at the bend opposite the pump (L1, Figure 1) was considerably lower than that along the long sides. On the contrary, in flumes containing gravel, the lowest velocities were measured often at location L3 but never at L1.

S3 – Counting procedure

Microplastic particles were counted with a Zeiss Stemi 2000 stereo microscope using bright light (1.6X magnification for fragments and 1.6-5.0X for fibers). Fragments were completely counted on all GF/D filters while fibers were counted completely only on GF/D filters with low fiber numbers only. Filters containing > 150 fibers were sub-sampled at 5.0X magnification in ten representative areas (about 6.25% of the entire filter area) and the cumulative fiber number was then upscaled to the whole filter area. Most filters containing fibers were counted twice by two different observers to account for observer bias and an average of both counts was used for further analysis. Fragment samples were counted only once by the same observer.

S4 – Calculations

The true sphericity index Ψ [-] of the nylon fibers was calculated following Cruz-Matías, et al. [6] using

$$\Psi = \frac{A_n}{A_{MP}} = \frac{\pi D_n^2}{2\pi r h + 2\pi r^2} \tag{S1}$$

with A_{MP} [L^2] as the true surface area of the fiber, A_n [L^2] as the nominal surface area of a sphere with the same volume as the fiber, $h = 500\ \mu\text{m}$ as the fiber length, $r = 7\ \mu\text{m}$ as the fiber radius, and D_n [L] as the nominal particle diameter of a sphere of equivalent volume. The Corey shape factor CSF [-] for the fibers was quantified following Dietrich [7] as

$$CSF = \frac{L_s}{(L_l L_i)^{1/2}} = 0.1673 \tag{S2}$$

with L_s, L_i, and L_l [L] as the shortest, intermediate and longest length of the fiber that are perpendicular to each other. Using the CSF we determined E [-], a geometric shape factor term contained in the Janke shape factor [7]. E relates L_s to the other two axes via

$$E = CSF\left(\frac{3}{\frac{L_l}{L_i}+\frac{L_i}{L_l}+CSF^2}\right)^{\frac{1}{2}} \tag{S3}$$

and was then used in equation (6) to calculate a modified V_s.

The flume-specific Reynolds number Re for open channel flow was determined as

$$\text{Re} = \frac{\varrho_w v R_h}{\mu} \tag{S4}$$

with ϱ_w [ML^{-3}] as the density of water, v [LT^{-1}] as the average flow velocity in in the flumes (Table S2), μ [ML^{-1}T^{-1}] as the dynamic viscosity of water (about 1.31 × 10^{-3} N·s/m^2 at 10°C) and R_h [L] as the hydraulic radius, which for an open rectangular channel can be determined by dividing its cross-sectional area by its wetted perimeter that in turn is determined by the water level height in the flume. The particle Reynolds numbers Re_P were determined using

$$\text{Re}_p = \frac{V_s}{\vartheta D_n} \tag{S5}$$

with V_s [LT^{-1}] as the particle settling velocity, ϑ [L^2T^{-1}] as the kinematic viscosity of water (about 1.3 mm^2 s^{-1} at 10°C), and D_n [L] as the nominal particle diameter that is based on mastersizer measurements for the fragments and SEM pictures and calculations for the fibers (see section S1).

Table S3 – Particle characteristics for nylon fragments and fibers.

				**Large fragment**	**Small fragment**	**Fiber**	**Comment**
Density of nylon used	ρ_{MP}	[kg/m^3]		1140	1140	1104	from manufacturer
Density of water	ρ_w	[kg/m^3]	1000				
Acceleration due to gravity	g	[m/s^2]	9.80665				
Kinematic viscosity	ϑ	[m^2/s]	1.31×10^{-6}				at 10° C
Dynamic viscosity	μ	[N s/m^2]	1.31×10^{-3}				at 10° C
Nominal particle diameter of sphere equivalent	D_{n1}	[μm]		516.40	237.54	52.78	of a sphere with equivalent volume
Particle diameter from mastersizer and SEM	D_{n2}	[μm]		478.63	181.97	14.00	SEM on fibers and Mastersizer on fragments
Dimensionless particle diameter of equivalent sphere	D_{*1}	[-]		110.17	10.72	0.09	Eq. (5)
Dimensionless particle diameter from mastersizer	D_{*2}	[-]		87.72	4.82		Eq. (5) using mastersizer measurements
Dimensionless settling velocity of equivalent sphere	w_{*1}	[-]		1.2381	0.0160	1.54×10^{-6}	Eq. (3) & (4)
Dimensionless settling velocity based on mastersizer	w_{*2}	[-]		0.8141	0.0035		Eq. (3) & (4) using mastersizer measurements
Stokes settling velocity	V_{s1}	[m/s]		0.0131	0.0031	1.27×10^{-4}	of equivalent spheres
Stokes settling velocity	V_{s2}	[m/s]		0.0114	0.0018	3.63×10^{-6}	using the diameters from measurements/calculations
Surface area	A_{MP}	[m^2]				2.23×10^{-8}	Eq. (S1)
Nominal surface area of sphere	A_n	[m^2]				8.75×10^{-9}	Eq. (S1)
True sphericity index	Ψ	[-]				0.3925	Eq. (S1)
Average diameter of fiber	d	[μm]				14	SEM, manufacturer
Length of fiber	L	[μm]				500	SEM, manufacturer
Corey shape factor	*CSF*	[-]				0.1673	Eq. (S2)
Janke shape factor	*E*	[-]				0.0484	Eq. (S3)
Average volume	V	[μm^3]		7.21×10^{7}	7.02×10^{6}	7.70×10^{4}	based on mass and density
Average mass	m	[g]		8.22×10^{-5}	8.00×10^{-6}	8.50×10^{-8}	for fragments based on laser diffraction, for fibers calculated

S5 – Modelling particle deposition and resuspension

In this work we used a derivation of the stochastic mobile-immobile model outlined by Roche, et al. [8], adapted for a recirculating flume. The model uses Continuous Time Random Walk (CTRW) theory. Particles are exchanged between a well-mixed water column (mobile zone) and the immobile zone sediment layer symbolizing a streambed. Particle concentrations are assumed to be spatially uniform in the water column. The following equation describes the concentration of particles, $C(t)$, in the well mixed water column:

$$\frac{dC(t)}{dt}V_f = -N_{dep}(t) + N_{res}(t) \tag{S6}$$

where V_f is the volume of water in the respective flume, while $N_{dep}(t)$ and $N_{res}(t)$ denote particle deposition onto and resuspension from the sediment layer, respectively [particles · s^{-1}]. $N_{dep}(t)$ can be described as a first-order boundary layer problem integrating the boundary layer flux of particles, J [particles/(m^2 s)], over the entire bed surface, A_{bed} [L^2] by

$$N_{dep}(t) = JA_{bed} \tag{S7}$$

Our assumption is that J is linked to the first-order removal rate, Λ [s^{-1}], and the concentration per unit bed area by:

$$J = \Lambda C(t)d \tag{S8}$$

with d as the flume-specific average water level [m] above the sediment bed. Thus

$$N_{dep}(t) = \Lambda C(t)dA_{bed} \tag{S9}$$

Adhesive, gravitational and other forces keep the deposited particles on the sediment bed until they are exceeded by shear forces at the bed surface (i.e., bed shear stress exceeds critical shear stress). These shear forces are caused mainly by turbulent flow moving over the bed. If we assume steady state flow conditions in the flumes, we can approach particle resuspension using a probability density function, $\varphi(t)$, to represent the probability of a particle resuspended at time t after deposition. The time a particle has remained immobilized since deposition $(t-\tau)$ and the number of particles deposited at the time of immobilization τ thus determine particle deposition as

$$N_{dep}(\tau) = \Lambda C(\tau)dA_{bed} \tag{S10}$$

while the probability of resuspension since the particle was immobilized is $\varphi(t-\tau)$. Total particle resuspension over the interval $t + \mathrm{d}t$ can then be calculated by integrating over all possible deposition times, $\tau \in [0, t]$:

$$N_{res}(t) = \int_0^t N_{dep}(\tau)\,\varphi(t-\tau)d\tau = \int_0^t \Lambda C(\tau) dA_{bed} \varphi(t-\tau)d\tau \qquad \text{(S11)}$$

Substituting equations (S10) and (S11) into equation (S6) yields

$$\frac{dC(t)}{dt} V_f = -\Lambda dA_{bed} C(t) + \Lambda dA_{bed} \int_0^t C(\tau)\varphi(t-\tau)d\tau \qquad \text{(S12)}$$

$$\frac{dC(t)}{dt} = \frac{\Lambda dA_b}{V_f}\left(-C(t) + \int_0^t C(\tau)\varphi(t-\tau)d\tau\right) \qquad \text{(S13)}$$

The coefficient dA_b is equivalent to the water volume above the sediment layer, V_R, and it follows $dA_b/V_f = V_R/V_f$. For a given initial concentration, C_0, at $t = 0$, we can solve equation (S13) in the Laplace domain and derive an analytical solution for $C(t)$. The Laplace transform of equation (S13) is:

$$u\tilde{C}(u) - C_0 = \frac{\Lambda dA_b}{V_f}\left(-\tilde{C} + \tilde{C}\tilde{\varphi}(u)\right) \qquad \text{(S14)}$$

where $\tilde{C}(u)$ and $\tilde{\varphi}(u)$ are the Laplace transforms of $C(t)$ and $\varphi(t)$, respectively. It then follows

$$\tilde{C}(u) = \frac{C_0}{u + \frac{\Lambda dA_b}{V_f}(1 - \tilde{\varphi}(u))}. \qquad \text{(S15)}$$

A power-law resuspension distribution $\varphi(t) \sim t^{-(1+\beta)}$, $(0 < \beta < 1)$ is assumed [9, 10]. In the Laplace domain it follows $\tilde{\varphi}(u) = 1/(1+u^{\beta})$, which can be substituted into (S14) to yield the Laplace-transformed analytical solution. We adapted a modified version of the MATLAB CTRW toolbox [11, 12] using DeHoog's algorithm [13].

We sampled the parameter space (n = 27,000 variations) using a Latin Hypercube approach. The balanced mean square error ($\hat{\theta}$) was used to assess performance [14]. It is defined as:

$$\hat{\theta} = \left(\frac{1}{n} \left[\frac{\sum_{i \subset n_A} \left(C_{sim,i}(\theta) - C_{obs,i} \right)^2}{(max(C_{obs}) - min(C_{obs}))^2} \right] + \left[\frac{\sum_{i \subset n_B} (log\ (C_{sim,i}(\theta)) - log\ (C_{obs,i}))^2}{(max(log\ (C_{obs})) - min(log\ (C_{obs})))^2} \right] \right)^{\frac{1}{2}}, \quad (S16)$$

where the total number of observations, n, is the sum of n_A and n_B, defined as the number of observations above and below a threshold concentration, respectively. A 20% of the peak threshold concentration has been shown to provide a balanced weight that considers all the breakthrough curve, including both the peak and tail [14, 15]. To test whether the parameter values corresponding to the lowest model errors were identifiable see e.g. [16] we plotted model error against parameter values for the top of the best-fits (i.e., lowest model error) and the best-fit model is presented with the data.

S6 – Microplastic particle counts

A total of 426 GF/D filters (18 blanks, 144 fiber filters, 264 fragment filters) from the three respective sampling locations in each flume were counted using light microscopy. Number concentrations of all three sampling locations per flume were then averaged, transformed to mass concentrations per flume volume and normalized to the respective flume-specific input concentrations. Of the 144 fiber filters, 2 were omitted as fibers were distributed very unevenly on the filter in large numbers and hard to count. Of the remaining 142 filters, 108 were counted twice by two different observers and averages of the two counts were used for further analysis where possible. Differences in counts by observer two as compared to observer one ranged from 0.01 to 118.42% (average of 20.50%; median of 15.56%). All 264 fragment filters were counted by one observer and all data could be used for further interpretation.

Table S4: Average microplastic particle counts (fibers and small fragments) in control flumes. No large fragments were found. G = gravel flume, M = mixed sediment flume, C = control flume.

Name	Setup	Samples	Fibers/20 mL	Fibers/47.5 L	Small fragments/20 mL	Small fragments/47.5 L
GC	gravel + water	6	1.83	4354.17	0.08	197.92
MC	mixed + water	6	1.75	4156.25	0.25	593.75
C	water	6	0.50	1187.50	0.30	712.50
Overall		18	1.36	3232.64	0.21	501.39

S7 – Microplastic particle concentrations

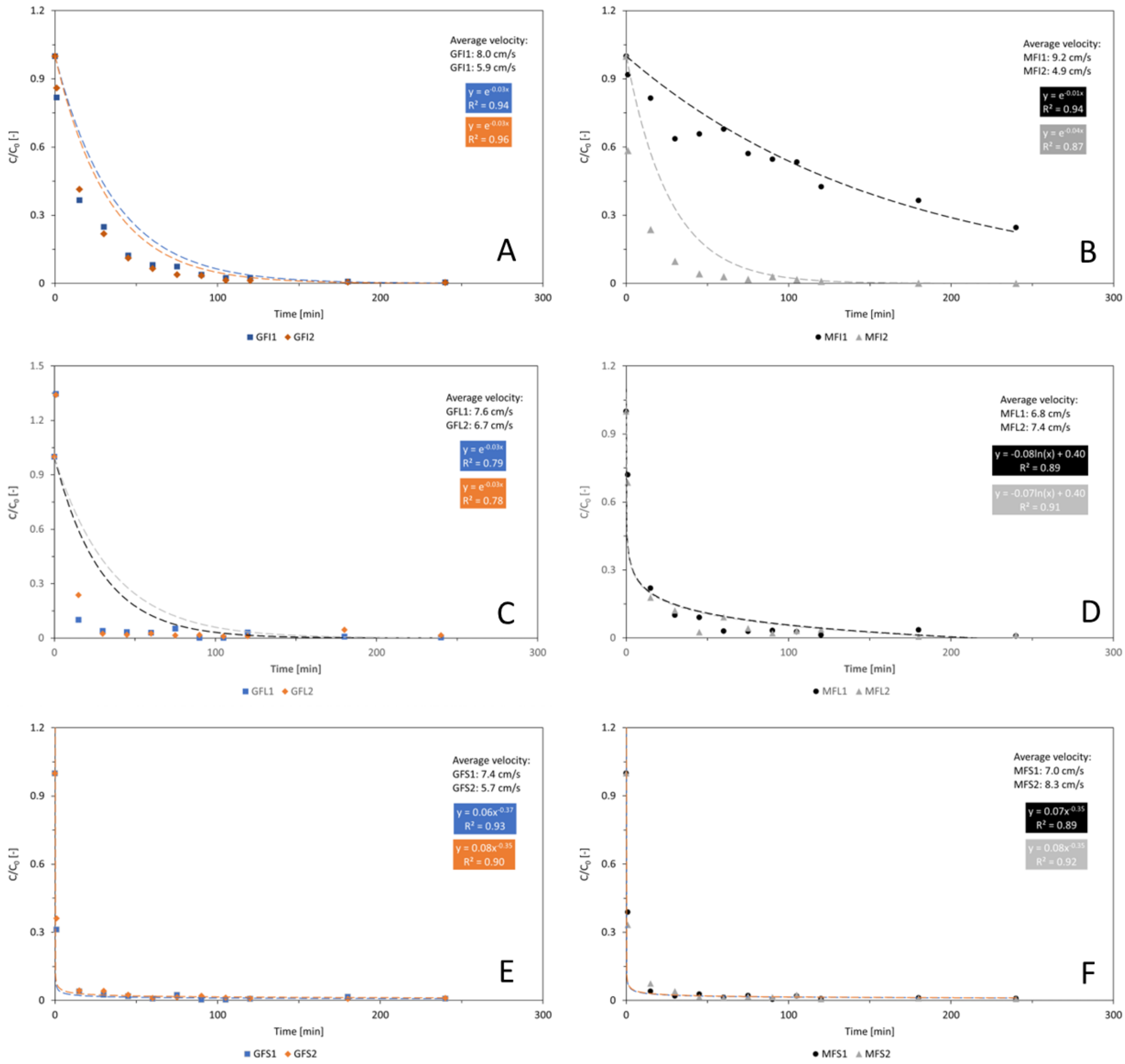

Figure S5: Normalized mass-based concentrations vs time for different flume setups. (A&B) Nylon fibers added to flumes containing gravel or mixed sediment. (C&D) Large fragments in flumes with gravel or mixed sediment. (E&F) Small fragments in flumes with gravel or mixed sediment. G = gravel flume, M = mixed sediment flume, FI = fiber flume, FL = large fragment flume, FS = small fragment flume. Flow velocities [cm/s] are always shown in upper right corners. R^2 values are based on trendlines (Flumes GFI, MFI and GFL follow exponential decay, flumes MFL follow a logarithmic decay and flumes GFS and MFS follow a power law function decay. Trendline equations are provided.

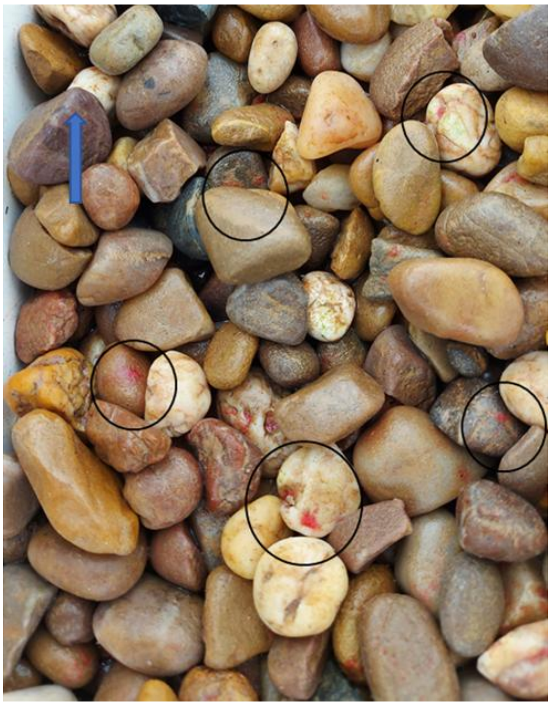

Figure S6: Flume GFI1 – Nylon fiber deposition on gravel sediment commonly occurs in microtopographic depressions on the sediment surface or at borders between gravel grains. Black circles indicate deposition areas, the blue arrow indicates the flow direction.

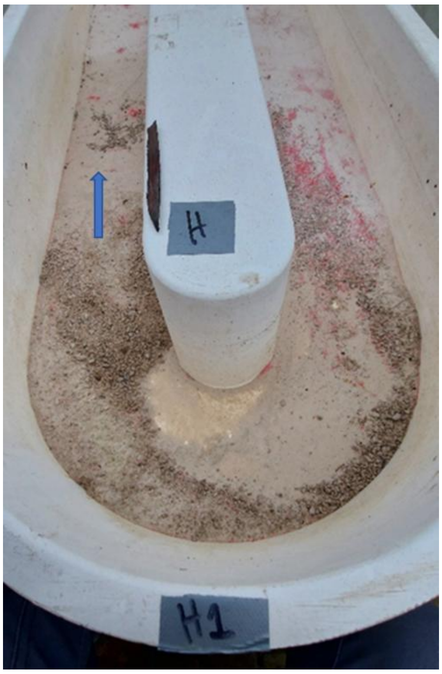

Figure S7: MFL2 - Most deposition of fragments seems to have occurred in patches along the long sides of the flume. Flumes often express changes in streambed microtopography as compared to the start of the experiment. At the bends, the sediment layer is visibly disturbed and has partially been redistributed, with little nylon deposited in that area. The blue arrow indicates the flow direction. Fragments were stained using Nile red prior to addition to the flumes to aid visualization.

S8 – Particle deposition

Table S5: Particle settling rates G_{MP} and expected times to complete particle settling assuming a static water column and linear settling. G = gravel flume, M = mixed sediment flume, FI = fiber flume, FL = large fragment flume, FS = small fragment flume.

Flume	Sediment	MP shape	Particle Settling Rates $[s^{-1}] \times 10^{-3}$		Expected Time to Complete Particle Settling [min]	
			A	B	A	B
GFI1	gravel	Fi	1.86	0.05	8.96	313.55
GFI2	gravel	Fi	1.89	0.05	8.83	308.96
MFI1	mixed	Fi	1.36	0.04	12.24	428.27
MFI2	mixed	Fi	1.29	0.04	12.89	451.21
GFL1	gravel	LF	191.10	166.18	0.09	0.10
GFL2	gravel	LF	179.70	156.27	0.09	0.11
MFL1	mixed	LF	142.46	123.88	0.12	0.13
MFL2	mixed	LF	143.50	124.79	0.12	0.13
GFS1	gravel	SF	42.78	25.77	0.39	0.65
GFS2	gravel	SF	45.53	27.43	0.37	0.61
MFS1	mixed	SF	32.27	19.44	0.52	0.86
MFS2	mixed	SF	33.44	20.15	0.50	0.83

SF = small fragments, LF = large fragments, Fi = fibers

A = For sphere of equivalent volume; B = For fragments based on mastersizer measurements, for fibers based on SEM and using Corey and Janke shape factors

Table S6: Average water levels, water flow velocities, hydraulic radii and open channel flow Reynolds numbers (Re) for the different flumes. G = gravel flume, M = mixed sediment flume, FI = fiber flume, FL = large fragment flume, FS = small fragment flume, C = control flume.

Flume	Average water level	Average velocity	Hydraulic radius	Re
	[m]	$[m\ s^{-1}]$	[m]	[-]
GFI1	0.068	0.0803	0.0358	2193
GFI2	0.067	0.0593	0.0355	1607
MFI1	0.093	0.0923	0.0416	2931
MFI2	0.098	0.0490	0.0425	1591
GFL1	0.068	0.0763	0.0358	2083
GFL2	0.073	0.0673	0.0369	1897
MFL1	0.092	0.0677	0.0413	2131
MFL2	0.091	0.0737	0.0411	2312
GFS1	0.072	0.0743	0.0366	2080
GFS2	0.067	0.0573	0.0355	1553
MFS1	0.095	0.0703	0.0419	2250
MFS2	0.092	0.0830	0.0413	2614
GC	0.061	0.0773	0.0336	1986
MC	0.096	0.0750	0.0420	2407
C	0.083	0.1223	0.0393	3672

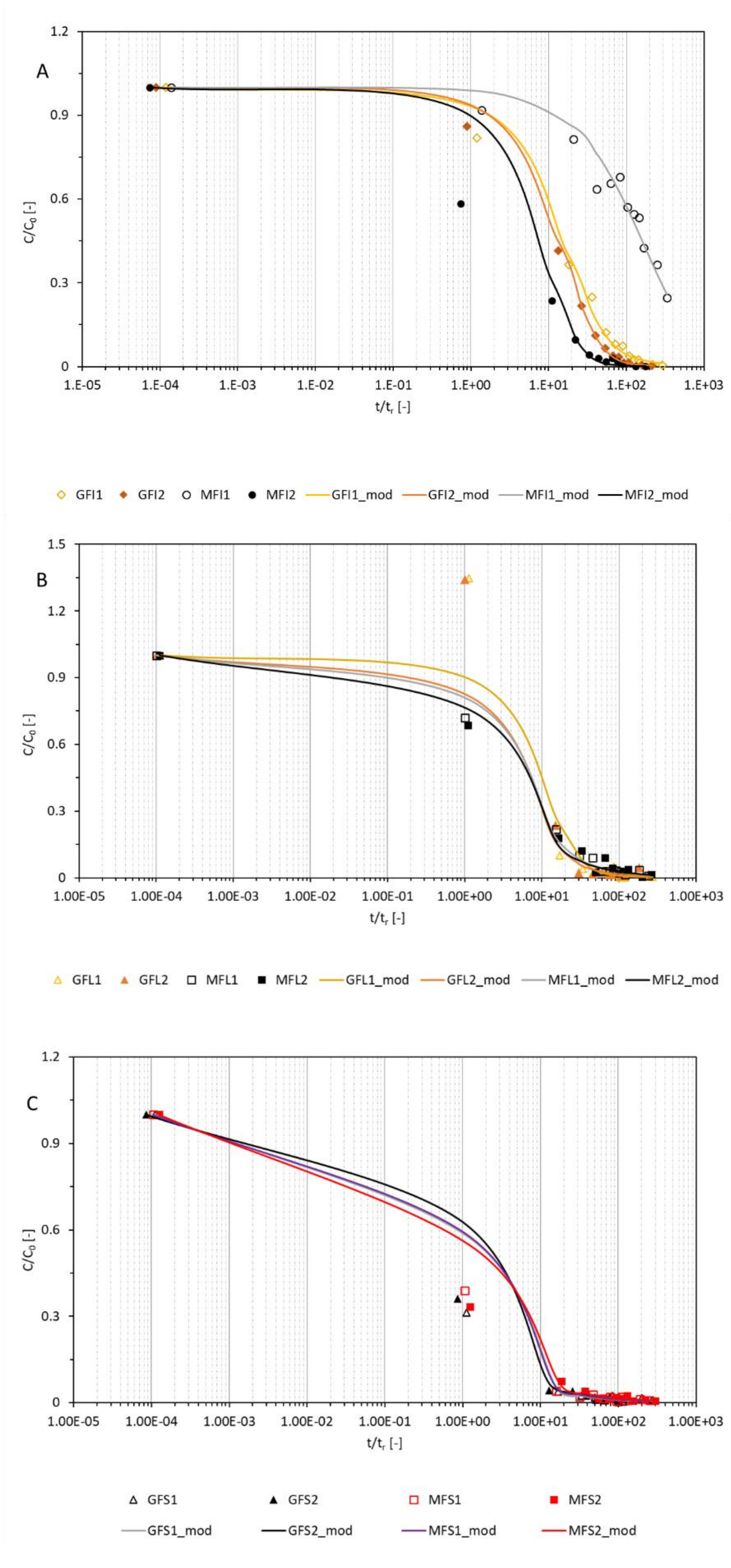

Figure S8: Modelled MP settling in the experimental flumes using the approach outlined in S5. A – Fiber flumes, B – Large fragment containing flumes, C – Small fragment containing flumes. Observed data is shown as individual measurements, modelling (mod) results are shown as continuous lines. G = gravel flume, M = mixed sediment flume, FI = fiber flume, FL = large fragment flume, FS = small fragment flume.

Table S7: Best fit parameters for the modelling results shown in Figures 4 and S8. They are based on 27,000 samples. STDEV = standard deviation based on values of all red dots in Figures S9-S11 (n=14). G = gravel flume, M = mixed sediment flume, FI = fiber flume, FL = large fragment flume, FS = small fragment flume.

Flume	Sediment	MP shape	MP size	Deposition Λ	STDEV	Resuspension β	STDEV
			[μm]	[s^{-1}]		[-]	
GFI1	gravel	fiber	500	0.132	0.007	0.153	0.008
GFI2	gravel	fiber	500	0.131	0.004	0.063	0.004
MFI1	mixed	fiber	500	0.021	0.002	0.222	0.015
MFI2	mixed	fiber	500	0.229	0.013	0.044	0.006
GFL1	gravel	fragment	large	0.195	0.014	0.085	0.007
GFL2	gravel	fragment	large	0.368	0.019	0.194	0.007
MFL1	mixed	fragment	large	0.402	0.039	0.265	0.015
MFL2	mixed	fragment	large	0.488	0.039	0.292	0.016
GFS1	gravel	fragment	small	0.974	0.028	0.248	0.012
GFS2	gravel	fragment	small	0.990	0.042	0.277	0.015
MFS1	mixed	fragment	small	0.987	0.037	0.277	0.011
MFS2	mixed	fragment	small	0.989	0.037	0.316	0.016

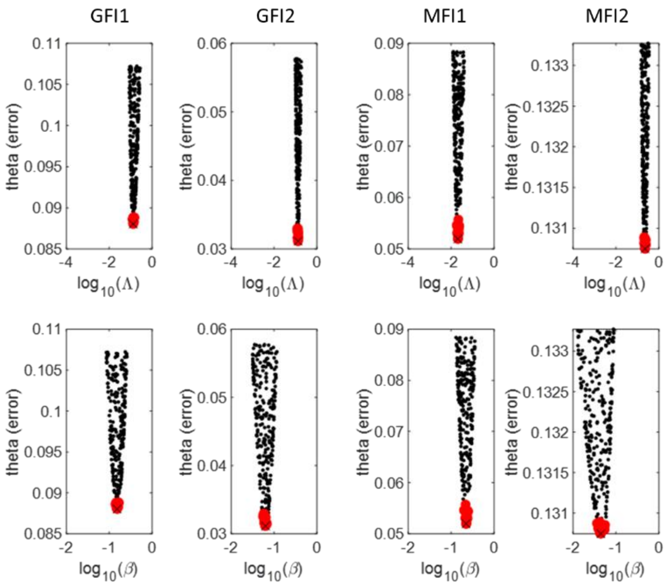

Figure S9: Model error vs. parameter values for fiber-containing flumes to demonstrate parameter sensitivity of best-fit model parameters shown in Figure S8 and Table S7 of in-stream measurements of MP. Black dots represent parameter values that meet a behavioral error threshold (top 1%) and red dots represent optimal parameter values (top 5% of the 1% shown or 0.05% of the total 27,000 runs). × symbolizes the best fit for the model curve shown in Figure S8, values are presented in Table S7. G = gravel flume, M = mixed sediment flume, FI = fiber flume.

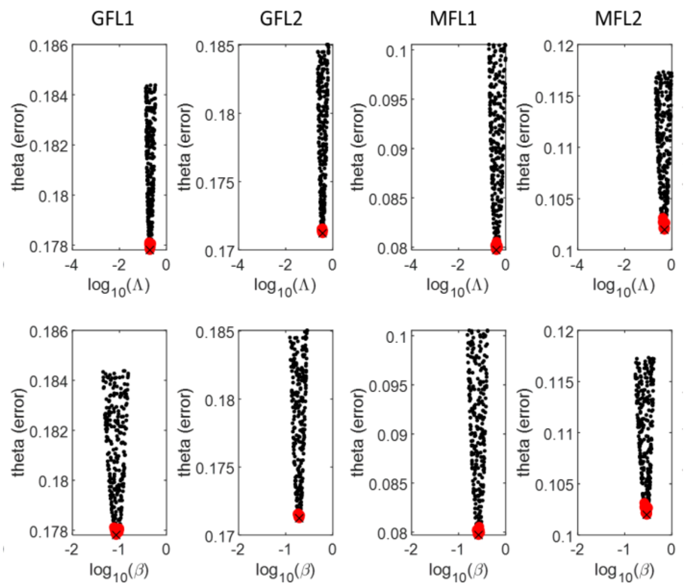

Figure S10: Model error vs. parameter values for large fragment containing flumes to demonstrate parameter sensitivity of best-fit model parameters shown in Figure S8 and Table S7 of in-stream measurements of MP. Black dots represent parameter values that meet a behavioral error threshold (top 1%) and red dots represent optimal parameter values (top 5% of the 1% shown or 0.05% of the total 27,000 runs). × symbolizes the best fit for the model curve shown in Figure S8, values are presented in Table S7. G = gravel flume, M = mixed sediment flume, FL = large fragment flume.

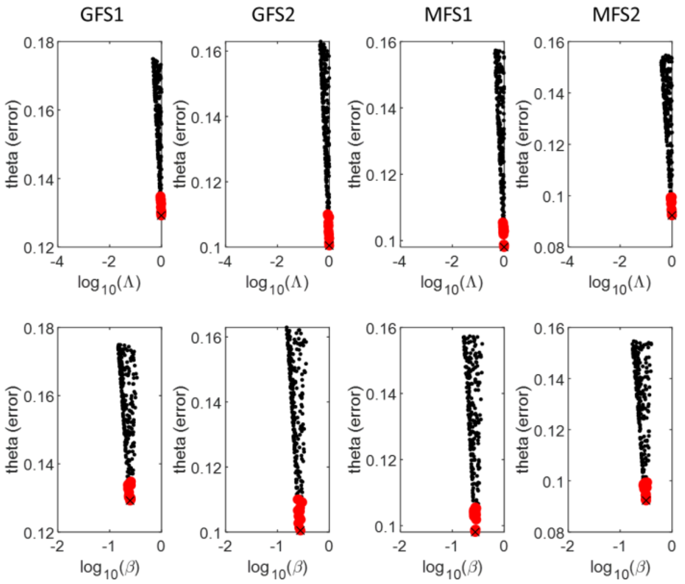

Figure S11: Model error vs. parameter values for small fragment containing flumes to demonstrate parameter sensitivity of best-fit model parameters shown in Figure S8 and Table S7 of in-stream measurements of MP. Black dots represent parameter values that meet a behavioral error threshold

(top 1%) and red dots represent optimal parameter values (top 5% of the 1% shown or 0.05% of the total 27,000 runs). × symbolizes the best fit for the model curve shown in Figure S8, values are presented in Table S7. G = gravel flume, M = mixed sediment flume, FS = small fragment flume.

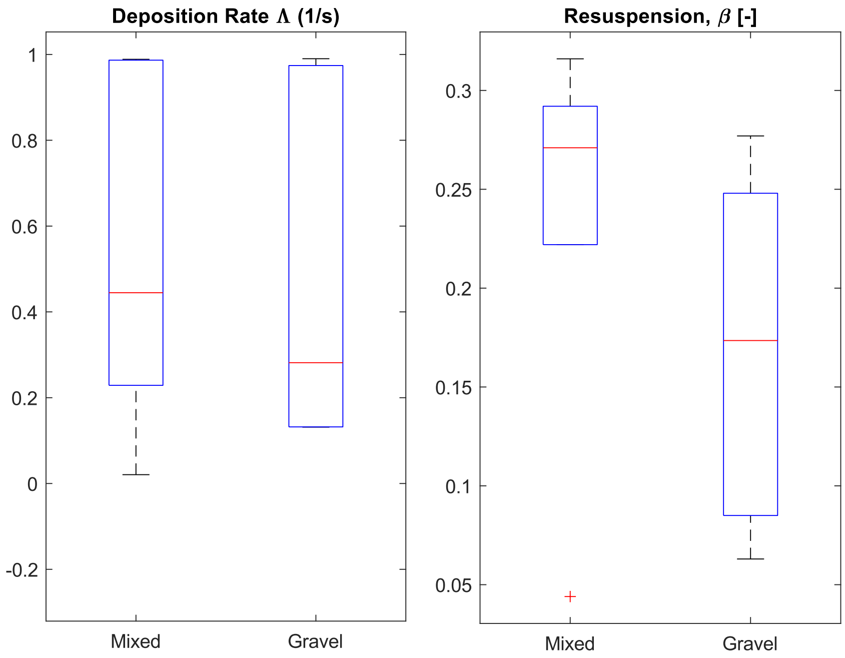

Figure S12: Box plots of deposition and resuspension rates grouped by sediment type (gravel, mixed) Statistics per group are based on best fit values (n=6, Table S7, × in Figure S8) for each flume. The red line symbolizes the median, blue box 25th and 75th percentiles, other lines show maxima and minima.

References

(1) Nel, H. A.; Chetwynd, A. J.; Kelleher, L.; Lynch, I.; Mansfield, I.; Margenat, H.; Onoja, S.; Goldberg Oppenheimer, P.; Sambrook Smith, G. H.; Krause, S. Detection limits are central to improve reporting standards when using Nile red for microplastic quantification. *Chemosphere* **2021**, *263*, 127953. DOI: 10.1016/j.chemosphere.2020.127953.
(2) Posselt, M.; Mechelke, J.; Rutere, C.; Coll, C.; Jaeger, A.; Raza, M.; Meinikmann, K.; Krause, S.; Sobek, A.; Lewandowski, J.; et al. Bacterial Diversity Controls Transformation of Wastewater-Derived Organic Contaminants in River-Simulating Flumes. *Environmental Science & Technology* **2020**, *54* (9), 5467-5479. DOI: 10.1021/acs.est.9b06928 (acccessed 2024/12/30/21:22:04). From DOI.org (Crossref).
(3) Jaeger, A.; Coll, C.; Posselt, M.; Mechelke, J.; Rutere, C.; Betterle, A.; Raza, M.; Mehrtens, A.; Meinikmann, K.; Portmann, A.; et al. Using recirculating flumes and a response surface model to investigate the role of hyporheic exchange and bacterial diversity on micropollutant half-lives. *Environmental Science: Processes & Impacts* **2019**, *21* (12), 2093-2108, 10.1039/C9EM00327D. DOI: 10.1039/C9EM00327D.
(4) Jaeger, A.; Posselt, M.; Schaper, J. L.; Betterle, A.; Rutere, C.; Coll, C.; Mechelke, J.; Raza, M.; Meinikmann, K.; Portmann, A.; et al. *Transformation of organic micropollutants along hyporheic flow in bedforms of river-simulating flumes*; 2021. DOI: 10.1038/s41598-021-91519-2.
(5) Arias Font, R.; Khamis, K.; Milner, A. M.; Sambrook Smith, G. H.; Ledger, M. E. Low flow and heatwaves alter ecosystem functioning in a stream mesocosm experiment. *Science of The Total Environment* **2021**, *777*, 146067. DOI: https://doi.org/10.1016/j.scitotenv.2021.146067.
(6) Cruz-Matías, I.; Ayala, D.; Hiller, D.; Gutsch, S.; Zacharias, M.; Estradé, S.; Peiró, F. Sphericity and roundness computation for particles using the extreme vertices model. *Journal of Computational Science* **2019**, *30*, 28-40. DOI: https://doi.org/10.1016/j.jocs.2018.11.005.
(7) Dietrich, W. E. Settling velocity of natural particles. *Water Resources Research* **1982**, *18* (6), 1615-1626. DOI: 10.1029/WR018i006p01615.
(8) Roche, K. R.; Drummond, J. D.; Boano, F.; Packman, A. I.; Battin, T. J.; Hunter, W. R. Benthic biofilm controls on fine particle dynamics in streams. *Water Resources Research* **2017**, *53* (1), 222-236. DOI: 10.1002/2016wr019041.
(9) Drummond, J.; Aubeneau, A.; Packman, A. Stochastic modeling of fine particulate organic carbon dynamics in rivers. *Water Resources Research* **2014**, *50* (5), 4341-4356.
(10) Drummond, J.; Davies-Colley, R. J.; Stott, R.; Sukias, J. P.; Nagels, J. W.; Sharp, A.; Packman, A. I. Retention and remobilization dynamics of fine particles and microorganisms in pastoral streams. *Water Research* **2014**, *66*, 459-472. DOI: http://dx.doi.org/10.1016/j.watres.2014.08.025.
(11) Cortis, A.; Berkowitz, B. Computing "anomalous" contaminant transport in porous media: The CTRW MATLAB toolbox. *Groundwater* **2005**, *43* (6), 947-950.
(12) Aubeneau, A. F.; Drummond, J. D.; Schumer, R.; Bolster, D.; Tank, J. L.; Packman, A. I. Effects of benthic and hyporheic reactive transport on breakthrough curves. *Freshw Sci* **2015**, *34* (1), 301-315. DOI: 10.1086/680037 (acccessed 2024/11/21).
(13) de Hoog, F.; Knight, J.; Stokes, A. An Improved Method for Numerical Inversion of Laplace Transforms. *SIAM Journal on Scientific and Statistical Computing* **1982**, *3* (3), 357-366.
(14) Bottacin-Busolin, A.; Marion, A.; Musner, T.; Tregnaghi, M.; Zaramella, M. Evidence of distinct contaminant transport patterns in rivers using tracer tests and a multiple domain retention model. *Advances in Water Resources* **2011**, *34* (6), 737-746. DOI: https://doi.org/10.1016/j.advwatres.2011.03.005.
(15) Riml, J.; Worman, A.; Kunkel, U.; Radke, M. Evaluating the fate of six common pharmaceuticals using a reactive transport model: Insights from a stream tracer test. *Science of the Total Environment* **2013**, *458*, 344-354. DOI: 10.1016/j.scitotenv.2013.03.077.

(16) Kelleher, C.; Wagener, T.; McGlynn, B.; Ward, A. S.; Gooseff, M. N.; Payn, R. A. Identifiability of transient storage model parameters along a mountain stream. *Water Resources Research* **2013**, *49* (9), 5290-5306. DOI: https://doi.org/10.1002/wrcr.20413 (acccessed 2024/09/30).
